# Family Size Evolution in *Drosophila* Chemosensory Gene Families: A Comparative Analysis with a Critical Appraisal of Methods

**DOI:** 10.1093/gbe/evu130

**Published:** 2014-06-19

**Authors:** Francisca C. Almeida, Alejandro Sánchez-Gracia, Jose Luis Campos, Julio Rozas

**Affiliations:** ^1^ Departament de Genètica and Institut de Recerca de la Biodiversitat (IRBio), Universitat de Barcelona, Spain; ^2^ Present address: CONICET, Departamento de Ecología, Genética y Evolución, Facultad de Ciencias Exactas y Naturales, Universidad de Buenos Aires, Instituto IEGEBA, Buenos Aires, Argentina.; ^3^ Present address: Institute of Evolutionary Biology, School of Biological Sciences, University of Edinburgh, Edinburgh, United Kingdom.

**Keywords:** chemosensory genes, gene birth-and-death, gene duplication, BadiRate, *D. sechellia*, gene tree–species tree reconciliation

## Abstract

Gene turnover rates and the evolution of gene family sizes are important aspects of genome evolution. Here, we use curated sequence data of the major chemosensory gene families from *Drosophila*—the gustatory receptor, odorant receptor, ionotropic receptor, and odorant-binding protein families—to conduct a comparative analysis among families, exploring different methods to estimate gene birth and death rates, including an ad hoc simulation study. Remarkably, we found that the state-of-the-art methods may produce very different rate estimates, which may lead to disparate conclusions regarding the evolution of chemosensory gene family sizes in *Drosophila*. Among biological factors, we found that a peculiarity of *D. sechellia*’s gene turnover rates was a major source of bias in global estimates, whereas gene conversion had negligible effects for the families analyzed herein. Turnover rates vary considerably among families, subfamilies, and ortholog groups although all analyzed families were quite dynamic in terms of gene turnover. Computer simulations showed that the methods that use ortholog group information appear to be the most accurate for the *Drosophila* chemosensory families. Most importantly, these results reveal the potential of rate heterogeneity among lineages to severely bias some turnover rate estimation methods and the need of further evaluating the performance of these methods in a more diverse sampling of gene families and phylogenetic contexts. Using branch-specific codon substitution models, we find further evidence of positive selection in recently duplicated genes, which attests to a nonneutral aspect of the gene birth-and-death process.

## Introduction

The chemosensory system of insects is composed of membrane receptors and ligand-binding proteins that belong to small to mid-sized (up to ∼300 genes) gene families (Benton et al. 2009; Nakagawa and Vosshall 2009; Sánchez-Gracia et al. 2009; Croset et al. 2010; Silbering and Benton 2010). Three of these families comprise chemoreceptor genes: the gustatory receptor (GR) family, which has been implicated in the recognition of soluble chemicals and CO_2_; the olfactory receptor (OR) family, which are responsible for the detection of airborne cues; and the ionotropic receptor (IR) family, which includes genes involved in both taste and olfaction. Phylogenetic analyses of GRs and ORs showed that these families are homologous to one another, with the latter being an insect-exclusive lineage evolved from the former (Robertson et al. 2003). Although insect ORs and GRs share some structural similarities with mammalian odorant receptors, some characteristics, such as a reverse orientation of the membrane topology, suggest independent origins of these genes in these taxonomic groups (Sato et al. 2008; Nakagawa and Vosshall 2009). In contrast, members of the IR family have a much older origin, with homologs detected in most animals (Croset et al. 2010). A chemosensory function, however, has been identified only for a subset of IRs and only in protostomes (Croset et al. 2010). Another mid-sized family involved in chemosensation in insects comprises the odorant-binding proteins (OBPs), small globular proteins that are widely expressed in the antennal sensillar fluid and are believed to mediate the interaction between odorants and receptors (Pelosi et al. 2006). Similar to the ORs, nonhomologous OBP molecules also exist in vertebrates (Tegoni et al. 2000).

The publication of genome sequence data from a large number of species has revealed that a considerable proportion of genes belong to gene families, particularly in eukaryotes (Rubin et al. 2000). However, as observed in the *Drosophila* chemosensory families, the number of family members is highly variable across genomes. Such variability accounts for a considerable proportion of genetic differences, even between closely related species, and constitutes an important source of phenotypic diversity (McLysaght et al. 2003; Fortna et al. 2004; Dumas et al. 2007). It has been proposed that gene family repertoires are determined by a dynamic process of gene gain and loss, which is thought to be mostly stochastic (Lynch and Conery 2003; Nei 2007; Demuth and Hahn 2009). Utilizing the gene birth-and-death model, differences in gene family size among species can be examined through two main parameters: the gene birth (gain) and death (loss) rates, which can be estimated using a well-resolved species tree with known divergence dates. Nevertheless, there are a number of methodological limitations that may preclude a systematic comparative analysis between gene families or taxonomic groups and that should be taken into account in such studies. The first and most obvious is the definition—or delimitation—of gene families and orthologous groups (OGs) (Hahn et al. 2007). Another major obstacle is the use of different estimation methods (with their particular assumptions), which can yield very different parameter estimates and, therefore, different biological interpretations. Species sampling can also affect the comparative analyses of gene families; because birth and death (BD) rates may change from one species or species group to another, estimates obtained using different species sets might not be comparable. Finally, because these analyses rely on evidence of gene presence and absence, the quality of whole-genome sequences and assemblies comprise another source of bias.

Chemosensation is essential for the detection and recognition of food sources, predators, and potential mates. Hence, this system constitutes one of the main mechanisms through which an animal interacts with the surrounding environment and is, therefore, highly adaptive. Recent studies have shown that the chemosensory gene families exhibit large differences in gene content across species, both in number and in subfamily composition (Sánchez-Gracia et al. 2009). For instance, the number of functional GR genes varies from 220 in *Tribolium castaneum* to 10 in *Apis mellifera* (Robertson and Wanner 2006; Engsontia et al. 2008). Large differences can also be found even among closely related species, such as *Drosophila simulans* and *D. sechellia,* which have 242 and 215 functional chemoreceptor genes, respectively. Such diversity makes the chemosensory gene families excellent subjects for the study of the molecular and evolutionary mechanisms underlying gene family evolution, gene duplication, functional novelty, and ecological adaptation. Some of the processes that have shaped the evolution of chemosensory gene sets have been extensively studied, albeit separately for each family and in different arthropod species or species sets (Robertson and Wanner 2006; Guo and Kim 2007; Vieira et al. 2007; Engsontia et al. 2008; Peñalva-Arana et al. 2009; Smadja et al. 2009; Croset et al. 2010). These studies have revealed a few general patterns that have been summarized in a number of review papers (Nei et al. 2008; Sánchez-Gracia et al. 2009). Overall, it has been shown that these families evolve according to the BD model, whereby new genes appear through duplication and are lost through deletion or pseudogenization (Nei and Hughes 1992; Nei and Rooney 2005). Another observed pattern is that most new genes arise through tandem gene duplication; thus, many recent paralogs are found in close proximity in genomes. Over time, these genomic clusters of paralogs will eventually be broken down by chromosomal rearrangements, although some genes may be maintained in a more clustered arrangement than expected by chance (Vieira et al. 2007; Vieira and Rozas 2011).

A comprehensive study of complete, well-annotated and curated, multigene families represents a great opportunity to analyze the major processes governing gene gains and losses and also to evaluate the impact of a number of methodological and biological factors affecting these analyses. In this study, we analyze the main gene families involved in the first steps of chemosensation in insects using information from 11 *Drosophila* genomes, with the objective of studying the evolutionary processes that govern the evolution of these gene families across closely related species of a well-studied taxonomic group. We applied, for the first time, a comparative framework using both the same methods and the same species set to study the four gene families. This framework allowed a comprehensive comparison of BD rates, both within and between gene families, and an evaluation of the impact of some biological processes that affect family size dynamics, such as ecology and demography, gene conversion, and the role of natural selection. Moreover, in our analyses of gene turnover rates, we explored different estimation methods using computer simulations to determine how comparable they are and which method produces the most accurate rate estimates for the *Drosophila* chemosensory gene families.

## Methods

### Data Sets

We used previously published data sets, some of which included manual gene (re)annotation and resequencing results that are not available in online databases. The OR sequences were provided by Guo and Kim (2007) (available on the website http://kim.bio.upenn.edu/software/dord.shtml, last accessed June 25, 2014), but the repertoire of each species was checked and corrected using the most recent updates of FlyBase and the resequencing results of Gardiner et al. (2008). The GR data set was kindly made available by M. Ritchie (data set used in Gardiner et al. 2008), and the OBP data set was that utilized in Vieira and Rozas (2011). The IR data set included data from Croset et al. (2010) (available as supplementary material for that paper). We performed some additional searches (using both BLAST and HMM [Hidden Markov Models] search methods) and manual reannotation on the IRs and found a few additional genes that were included in the analyses presented herein.

### OG Identification

The accurate identification of orthologous and paralogous genes is critical for estimating gene BD rates using methods that take into account information on gene orthology (see below). The most reliable way of delimiting OGs is by phylogenetic inference (Gabaldon 2008); however, phylogenetic methods are very sensitive to the quality of the sequence alignment, which can be problematic, particularly in large gene families or when gene sequences are too divergent. We attempted to overcome the OG identification problem by combining clustering techniques based on sequence similarity (BLAST algorithm) with phylogenetic methods using a semiautomated pipeline (written in *AWK* and Perl). In a first step, we obtained major gene clusters based on amino acid sequences with CLANS (Cluster Analysis of Sequences; Frickey and Lupas 2004) using a specific cutoff *E* value for each family (chosen empirically to provide a good number of clusters while minimizing singletons). In this way, we preselected sequences (in clusters) to be aligned with each other, avoiding the alignment of very divergent sequences. The multiple alignment of sequences (MSA) in each cluster was obtained with MAFFT (Katoh et al. 2005); these MSA were then used for building phylogenetic trees with the program RAxML version 7 (Stamatakis 2006) using full likelihood searches (-f d option) and the *PROTGAMMAWAG* substitution model. We then determined the orthology assignments based on reconciliation between the clades found in these trees and the accepted *Drosophila* species phylogeny (Clark et al. 2007). We defined an OG as the most inclusive group (clade on the tree) compatible with the *Drosophila* species tree. To facilitate this step, we wrote a Perl script that uses the cluster tree as input and retrieves the sequences included in each clade representing an OG based on a cutoff branch length that was empirically chosen to meet our OG criterion. A few clades, however, were represented by sequences of only one *Drosophila* subgenus; these clades were double-checked (phylogenetically and by BLAST) to ensure that they constituted a separate OG (i.e., the members of this OG were lost in one of the subgenera). This approach was generally straightforward, with a few exceptions in the OBP family, as discussed in the Results section.

### Gene Conversion

Interlocus gene conversion is a nonreciprocal transfer of genetic information in which a sequence fragment of one paralog is pasted into the homologous gene region of the other paralog (Petes and Hill 1988; Osada and Innan 2008; Ohta 2010). As a result, fragments of the paralogs’ sequences will be more similar to each other than expected given the time since the gene duplication event. Gene conversion events can lead to errors in the phylogenetic reconstructions of gene trees and, therefore, may bias gene BD rate estimates based on gene tree–species tree (GT-ST) reconciliation. We used the GENCONV software (Sawyer 1989) to assess whether chemosensory paralogous gene pairs had undergone gene conversion. This program identifies gene conversion events by detecting putative sequence fragments that appear to have been transferred from one paralog into another (i.e., fragments in a multisequence alignment that have higher sequence similarity to a homologous paralog fragment than would be expected based on the mean similarity levels across the entire gene). We applied the global inference option, which calculates the probabilities using multisequence alignment of the entire OG.

### Gene Birth and Death Rates: GT-ST Reconciliation

We manually mapped the gene duplication and loss events on the phylogeny by performing GT-ST reconciliation separately for each OG. This method uses the parsimony principle to fit gene lineages into species lineages by identifying gene duplication and loss events that cause observed differences between an OG gene tree and the species trees (Goodman et al. 1979). Amino acid sequences were aligned using MAFFT, and gene trees were obtained with RAxML as described earlier. To overcome common biases related to poorly resolved phylogenies (Hahn 2007), we used an approach similar to that described as the species-overlap method (Gabaldon 2008). When faced with disagreement between the gene and species trees, we used a conservative criterion that takes into account short branch lengths and the known problems of incomplete lineage sorting that lead to inconsistencies across genes in the position of *D. willistoni* (Tamura et al. 2004; Obbard et al. 2012) and the relationships among *D. yakuba*, *D. erecta*, and the *melanogaster* cluster (Pollard et al. 2006). For instance, if there is only a single *D. willistoni* gene but it was clustered with the species of the *Drosophila* subgenus, we assumed an error in the tree reconstruction and, therefore, did not count any events. Thus, we mostly counted duplications and losses if there was more than one sequence per species or if one or more species were missing from the OG. We also applied a conservative criterion if a single species had several copies of the same OG in a poorly resolved phylogeny; in this case, we favored the placement of gene duplications at the tips, thereby avoiding overestimation of gene losses.

After inferring the number of gene duplications and losses and the total family size in each internal node of the phylogeny, we estimated the global gene birth (β) and death (δ) rates in two ways. First, we applied equations (1) and (2) from Vieira et al. (2007) to obtain a rate based on the proportion of gains and losses per branch and the time since the origin of the clade under study (GT-ST Rec). Second, we used modified versions of those equations in which BD rates are obtained for each branch and then averaged across all branches (GT-ST Rec-Av):

(1) $$\beta =\frac{\sum_{i=1}^{n}\left[Gi/\left(Ci*Ti\right)\right]}{n},$$

(2) $$\delta =\frac{\sum_{i=1}^{n}\left[Li/\left(Ci*Ti\right)\right]}{n},$$

where *n* is the number of branches in the species phylogeny, *Gi* and *Li* are the number of gains and losses in branch *i*, respectively; *Ci* is the number of genes at the ancestral node of branch *I*; and *Ti* is the time length of branch *i* (in Myr). In this way, the global estimates are more sensitive to heterogeneity in the branch rates and more comparable to the results of the full maximum-likelihood methods that take branch lengths into account (see below). We dubbed this approach “branch average.”

### Gene Birth and Death Rates: Fully Automated Methods

The BD rates were also estimated using fully automated methods with the program BadiRate (Librado et al. 2012) version 1.3. We applied four different methods implemented in BadiRate to obtain β and δ estimates. The first method, BadiRate CSP, uses a modification of the Sankoff parsimony algorithm to estimate the family size in each internal node, which is then used to determine the number of gains and losses per branch (as the difference of the number of copies between ancestral and derived lineages). The second method, BadiRate CWP, is similar, with the difference that it uses Wagner parsimony instead. The third method, BadiRate CML, is similar to BadiRate CSP but estimates the family size in each internal node by maximum likelihood. All three methods then employ the same equations used in the GT-ST Rec method (eqs. 1 and 2 from Vieira et al. [2007]) to calculate rates. With the BadiRate CWP method, we additionally employed the “branch average” approach using the novel equations herein proposed (BadiRate CWP-Av). These methods use OG information as the GT-ST reconciliation method. In this way, the input consists of the number of genes per species per OG.

The third and fourth methods, BadiRate BD-GR-ML and BadiRate L-GR-ML, use a full maximum-likelihood approach to determine the rates that maximize the probability of observing the total number of genes per species (see BadiRate documentation for further information). These methods use as input the total gene count per species (as opposed to having gene counts separated by OG). The maximum-likelihood framework allows them to take into account undetected BD events (duplicated genes that were later lost without leaving evidence) and also allows hypothesis testing by model comparison. The difference between the methods is that BadiRate L-GR-ML assumes that β and δ are identical, instead estimating λ, a general measure of gene turnover rate. Because BadiRate BD-GR-ML and BadiRate L-GR-ML are likelihood-based and, therefore, subject to problems of entrapment at local optima, we ran 100 replicas using different starting seeds (*-start_val 1* option, seeds provided by a random number generator). We considered that convergence was achieved when the lowest likelihood value was clearly overrepresented among 100 independent runs. Both ML methods assume that all new genes appear through gene duplication as opposed to innovation (de novo gene origin). The BadiRate BD-GR-ML and BadiRate L-GR-ML methods are very similar to those available in the program CAFE (De Bie et al. 2006), which we did not use to avoid redundancy. The commands used in the BadiRate analyses are listed in the supplementary methods, Supplementary Material online.

**Fig. 1.—:**
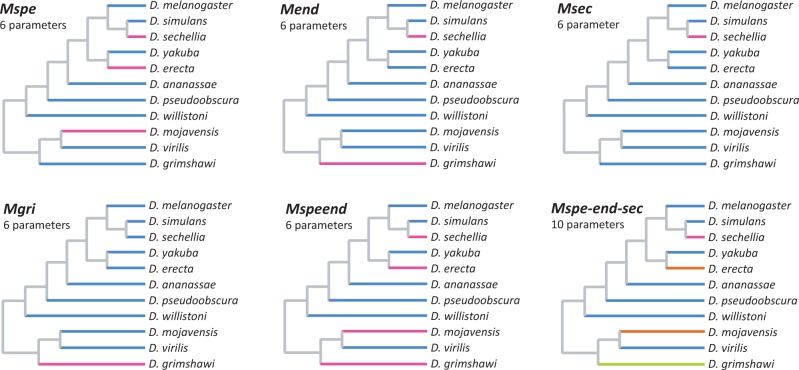
Branch models used to evaluate rate differences among species. In each tree, branches in the same color were set to have the same rates and were allowed to have rates different from those of the branches in different colors.

To test the effect of different ecological peculiarities (diet specialization and endemism) on gene turnover rates, we used the *–bmodel* option of BadiRate (BD-BR-ML model), which allows the assumption of different BD rates for prespecified lineages. We assumed, for simplicity, that all the internal branches of the phylogeny shared the same BD rates (i.e., given that we cannot establish the ecology of the ancestral species, the BD rates of chemosensory families in internal branches were considered as nuisance parameters), whereas the terminal branches were allowed to either share or have their own turnover rates, depending on their ecology. In this way, we explored eight scenarios (branch models [BMs]) based on the distinctiveness of the BD rates of the following species groups: Diet specialists (*D. sechellia*, *D. erecta*, and *D. mojavensis*; model *Mspe*), endemics (*D. sechellia* and *D. grimshawi*; model *Mend*), specialist or endemic (*D. sechellia*, *D. erecta*, *D. mojavensis*, and *D. grimshawi*; *Mspeend*), specialist and endemic (*D. sechellia*; model *Msec*), and only endemic (*D. grimshawi*; model *Mgri*). All these models have six lineage-specific parameter rates (three β and three δ): β and δ of the internal branches, β and δ of the terminal focal branch(es), and β and δ of the remaining terminal branches (fig. 1). Additionally, we also assumed a more complex scenario in which we included all the specialists and endemics but with separate rates for the specialists (*D. erecta* and *D. mojavensis*), endemics (*D. grimshawi*), and *D. sechellia*, as it meets both criteria (model *Mspe-end-sec*, a total of ten rate parameters; fig. 1). Finally, we explored the Global rate model (*MGr*) in which all terminal branches shared the same β and δ and the Free rate model (*MFr*) in which each terminal branch may have different rates. All the aforementioned BMs were analyzed separately for each gene family and also for a data set that included all the chemosensory families. The BM analyses were performed in 100 independent runs using different random starting values. We compared the goodness of fit of these models using Akaike Information Criterion (AIC; Akaike 1973).

Throughout the study, we used the *Drosophila* species divergence tree and divergence dates proposed by Tamura et al. (2004); rates are expressed in units of the number of events per gene per million years. All the BD rate estimates were performed using the *Drosophila* genomes reported in Clark et al. (2007), with the exception of *D. persimilis*. We excluded this species because 1) its genome has the lowest coverage among those studied herein; 2) the branch length connecting this species to *D. pseudoobscura* is very short and, therefore, there is a considerable amount of incomplete lineage sorting; 3) there is ongoing gene flow between *D. persimilis* and *D. pseudoobscura* (Kulathinal et al. 2009); and 4) there was no double checking of frameshifts and stop codon mutations through resequencing (OR and GR) as had been done for *D. sechellia* and *D. simulans* (Gardiner et al. 2008). These factors can severely affect BD rate estimates.

### Computer Simulations

To gain insight into the performance of the different methods to estimate BD rates in the *Drosophila* chemosensory gene families, we ran two simulation experiments. In experiment 1 (Exp. 1), we evaluated the performance of the different estimation methods in gene families where all branches of the phylogeny have identical rates, whereas in experiment 2 (Exp. 2), we compared these methods in a situation where the amount of rate heterogeneity among lineages is similar to that detected in *Drosophila* chemosensory families (i.e., one species out of eleven has a distinctive turnover rate; table 1). In both experiments we simulated families with 50 OGs each (within the range observed among the *Drosophila* chemosensory gene families), where each OG had one gene copy at the root of the species tree and evolved independently from the other OGs. Most simulations for Exp. 1 and Exp. 2 were performed with a Perl script (kindly provided by P. Librado), which adapts the stochastic models implemented in the software BadiRate (Librado et al. 2012) for simulations and that will be included in the next version of this program. These experiments were carried out over 500 simulation replicates at two rate magnitudes (low: β = δ = 0.002 events/gene copy/Myr and high: β = δ = 0.02 events/gene copy/Myr).

**Table 1:** Birth (β) and Death (δ) Rates Simulated in Exp. 2

Simulation	Branch Rates			
	Internal (β = δ)	Background (β = δ)	Foreground (β)	Foreground (δ)
*sBRlow5*	0.01	0.002	0.002	0.01
*sBRlow10*	0.01	0.002	0.002	0.02
*sBRhigh5*	0.01	0.02	0.02	0.1
*sBRhigh10*	0.01	0.02	0.02	0.2

Estimates of BD rates for each replicate were obtained with the BadiRate BD-GR-ML, BadiRate CML, BadiRate CSP, BadiRate CWP, and BadiRate CWP-Av methods. Because the Perl script used in the simulation experiments produces neither sequences nor trees (i.e., the output of the simulation script is a list with the total number of gene copies of an OG in each species), it was not possible to evaluate the GT-ST reconciliation procedure using those simulations. To assess the performance of the GT-ST Rec methods and compare them with the other methods, we used the package HyPhy for R (Hallinan 2013) that produces simulated gene family trees. Because the GT-ST Rec analysis is not automated and, hence, time-consuming, we simulated and analyzed only ten gene families for each rate magnitude as in Exp. 1. Although ten replicas is a very low number for a simulation experiment it should be a good compromise instead of not evaluating the GT-ST Rec method at all. In all simulation experiments, the estimation error was calculated as the normalized Euclidean distance between estimated and simulated BD rates (i.e., the square of the difference between the observed and the simulated rates, divided by the simulated rate). See supplementary methods, Supplementary Material online, for further details on the simulation experiments.

### Selective Constraints on Duplicated Genes

We studied the role of selection in the evolution of duplicated chemosensory genes by using likelihood-based model comparisons and the parameter ω (ω = *d*_N_/*d*_S_, where *d*_N_ and *d*_S_ are the nonsynonymous and synonymous substitution rates, respectively), a measure of the selective constraint acting on coding sequences. The evolutionary model we evaluated assumed two classes of branches with different ω values: Duplication branches (resulting from a duplication event labeled on the OG’s phylogenetic tree) and speciation branches (the remaining branches, i.e., those resulting from speciation events). For these analyses, we first built MSA of the amino acid sequences for each OG using MAFFT and then used those MSA to guide the corresponding nucleotide sequence alignments using the Perl script pal2nal.pl (Suyama et al. 2006). Next, we identified and labeled gene duplication events on the OG phylogenetic trees obtained from the amino acid sequence data. The nucleotide alignments and labeled trees were used as input for the BM analysis (*model* = 2) implemented in the program *codeml* of the PAML 4 package (Yang 1997, 2007). The statistical significance of the ω differences between the two classes of branches was obtained by comparing the fit of the BM with that of a null model (M0), in which all lineages have the same ω, using the likelihood-ratio test (LRT). This test is quite conservative because all duplicated branches are assumed to share a particular ω value, whereas the prediction is that one (not necessarily both) of the duplicated copies is under relaxed selective pressure (Ohno 1970; Pegueroles et al. 2013). To avoid problems with sequence saturation, we excluded OGs with *d*_S_ > 2 from the analyses. Moreover, we also excluded 1) all OGs with a very large number of duplications, 2) all duplicates with incomplete or dubious gene annotation, 3) very recent duplications (with zero or close to zero *d*_N_ values), and 4) pseudogene sequences. The sequential Bonferroni correction was applied to correct the alpha level for multiple tests. To test for the presence of positively selected sites in four OGs with elevated ω values, we applied the branch-site approach of PAML (used *model = 2* and *nsites = 2*). The statistical significance of the model was determined upon contrast with a null model in which ω was fixed at a value representing neutral selection (*ω = 1*).

## Results

The number of OGs identified per family varied from 47 to 58 (supplementary tables S1–S4, Supplementary Material online). Four OGs of the OBP family were present only in the *D. melanogaster* group, with three of them found only in the *D. melanogaster* subgroup. These four OGs were excluded from the GT-ST Rec analyses (see Discussion). A preliminary assessment of the gene BD rates estimated for the four *Drosophila* chemosensory gene families showed important differences among methodologies (supplementary fig. S1 and tables S5 and S6, Supplementary Material online). Thus, we decided to investigate whether these families present two features that could potentially cause bias in turnover rate estimation: Rate heterogeneity among lineages and gene conversion. Subsequently, to address the methodological factor affecting the observed differences, we compared the performance of the different rate estimation methods using computer simulations.

### Heterogeneity in Gene Turnover Rates among Lineages

Using a maximum-likelihood approach, we compared several models of rate heterogeneity among lineages. For the three membrane receptor families, the model that best fit the data was the *Msec* model (table 2 and supplementary table S7, Supplementary Material online), indicating that the gene turnover rates of *D. sechellia* are significantly different from those of the other species analyzed. In contrast, the best-fitting model for the OBP family was *Mspeend* (in which specialists and endemics share a distinctive rate), although the AIC differences between this and other models (*Mspe* and *MGr*) were quite small and nonsignificant (table 2). To improve the statistical power, we performed the same analysis combining all four gene families (table 2). Again, the results favored the *Msec* model, with a large advantage in terms of AIC. The lineage rate estimates showed that *D. sechellia* had, in general, much higher δ than β (supplementary table S7, Supplementary Material online) and that the *D. sechellia*’s δ estimates were also much higher than the δ estimates of the other species analyzed. It is important to notice that we could not compare the fit of the most complex models because convergence of the likelihood values was never achieved in any of the gene families, even after 100 independent runs. It is possible, therefore, that the *Mspe-end-sec* and *MFr* models would actually fit the data better than the simpler *Msec* model. In any case, our analyses suggest a strong effect of *D. sechellia* in the interlineage variation of gene turnover rates.

**Table 2:** AIC Values for Different BMs of Gene Turnover Rates Obtained with BadiRate BD-BR-ML (*-bmodel* Option)

Model^a^	np	OBP	OR	GR	IR	All Families
*MGr*	2	71.06	80.60	89.68	90.80	324.25
*Msec*	6	74.28	**69.67**	**82.39**	**73.68**	**292.19**
*Mgri*	6	73.09	84.84	90.53	90.89	324.67
*Mspe*	6	71.05	78.91	86.37	91.21	317.16
*Mend*	6	72.96	75.13	86.67	86.86	309.02
*Mspeend*	6	**70.91**	80.85	88.91	93.46	323.64
*Mspe-end-sec*	10	n/a	n/a	n/a	n/a	326.77^b^

Note.—The best AIC value for each family is in bold letters. np, number of parameters; n/a, not applicable.

^a^See text for a description of the models.

^b^Lowest local maximum obtained after 100 runs; far from convergence.

### Gene Conversion Effect on the Estimation of BD Rates

We analyzed the presence of the hallmark of gene conversion in 14 OGs with complex GT-ST reconciliation (supplementary table S8, Supplementary Material online). Although gene conversion between paralogs has a high potential to bias the estimates of gene turnover rates based on GT-ST reconciliation, its impact will largely depend on its effect in the gene tree reconstruction (i.e., the gene conversion tract has to be large enough to blur the phylogenetic information contained in the nonaffected parts of the gene; see supplementary methods, Supplementary Material online, for explanation). Although we detected a high probability of gene conversion in eight OGs (supplementary table S8, Supplementary Material online), most of these putative gene conversion events would not pose a problem for β and δ estimation, as they did not appear to affect the gene tree inference (the gene conversion test may give false positives if duplications are very recent or different parts of the gene are under different selective constraints). However, in two IR OGs, as gene conversion possibly affected the gene tree topology, we took this into account when mapping duplications and losses onto the gene tree (supplementary methods, Supplementary Material online). Furthermore, there were some putative gene conversion cases in which it was not clear whether gene tree reconstruction had been affected (three IRs and one GR—the “maybe” instances in the supplementary table S8, Supplementary Material online). We attempted to predict how gene conversion would affect gene tree reconstruction for the four OGs with “maybe” instances and re-estimated the gene BD rates using the GT-ST Rec method (not counting pseudogenes) for divergent IRs (β = 0.0025, δ = 0.0030) and GRs (β = 0.0049, δ = 0.0026). These new estimates were very similar to those obtained without considering gene conversion events (IR: β = 0.0030, δ = 0.0031 and GR: β = 0.0050, δ = 0.0026), suggesting that overall the effects of gene conversion on the rate estimates of the gene families analyzed herein were minimal.

### Simulation Experiments

**Fig. 2.—:**
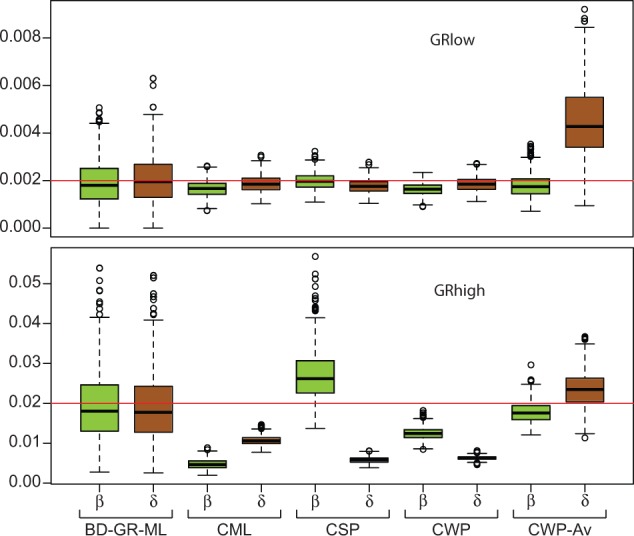
Global birth (β) and death (δ) rate estimates across the simulated gene families of Exp. 1, using different estimation methods. Red line marks the expected (simulated) values. See main text for details.

Because of the discrepancy among BD rate estimates obtained with different methods in the preliminary analysis (supplementary table S5, Supplementary Material online), we used computer simulations to evaluate the performance of these methods when applied to gene families similar to the *Drosophila* chemosensory families and with known turnover rates. In Exp. 1, we evaluated methods in the simplest case where all branches of the phylogeny share the same BD rates (fig. 2 and supplementary fig. S2, Supplementary Material online). Although the analyses based on simulated gene trees used only ten replicates per rate tested, the results are in general agreement with the results based on 500 replicates (supplementary figs. S3 and S4, Supplementary Material online). At low rates (0.002), the methods that use the Vieira et al. (2007) equations and OG information (BadiRate CML, CSP and CWP, and GT-ST Rec) showed good precision (reproducibility) and accuracy (proximity to the true rate). On the other hand, these methods were very inaccurate at high turnover rates (0.02), in which case they tended to underestimate rates (i.e., precisely 1.5- to 2-fold with the GT-ST Rec method). The BadiRate CWP-Av and the GT-ST Rec Av methods performed poorly in estimating death rate at low rates, but behaved better at high rates. The BadiRate BD-GR-ML presented the lowest precision in estimates at both simulated rates, even though it was accurate on average across replicates.

**Fig. 3.—:**
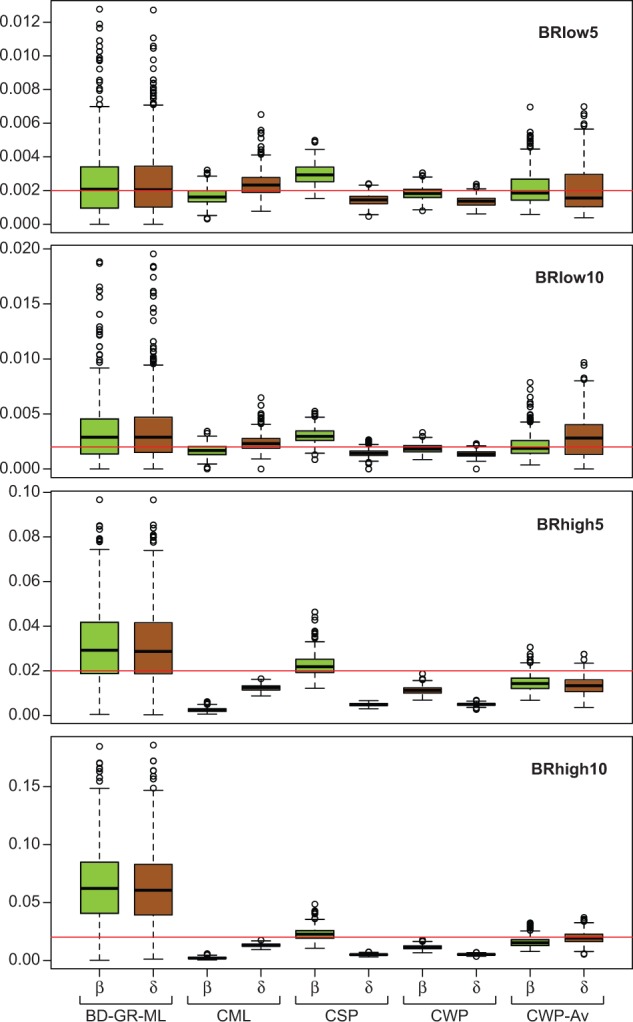
Global birth (β) and death (δ) rate estimates for the simulated gene families of Exp. 2.1, using different estimation methods. Red line marks the expected (simulated) values. See main text for details.

**Fig. 4.—:**
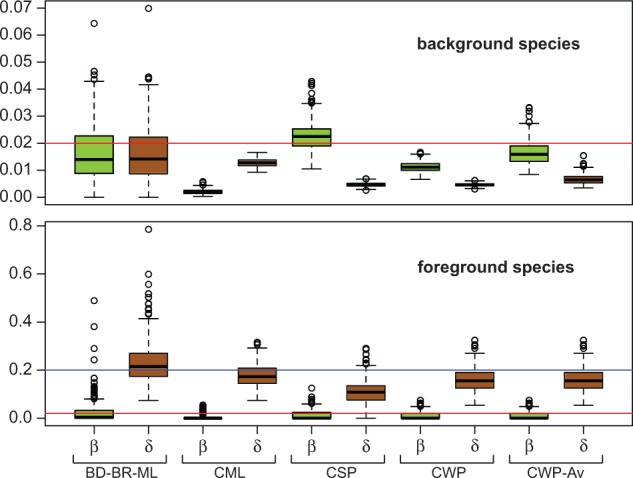
Gene birth (β) and death (δ) rate estimates obtained for the foreground and background species in 410 *BRhigh10* simulations of Exp. 2.2 (see text and table 1 for details), in which significant rate differences among species were detected. Red lines mark the expected (simulated) value 0.02 (β = δ of background species and β of foreground species) and the blue line marks the expected value 0.2 (δ of the foreground species).

In the Exp. 2.1, we simulated rate heterogeneity among species similarly to the observed in the empirical data, but analyzed the simulated data disregarding such heterogeneity (see supplementary methods, Supplementary Material online). The results for low rate simulations were very similar to the ones obtained in the Exp. 1 (BadiRate CWP and CSP had the best performance), except for an important reduction in the precision of the estimates (fig. 3 and supplementary fig. S5, Supplementary Material online). The BadiRate BD-GR-ML method was considerably more affected by the presence of a lineage with a different death rate than the OG-based methods, independently of the magnitude of the BD rates across branches; a larger difference between simulated background and foreground rates (*BRlow10* and *BRhigh10*) exacerbated the problem. At high rates, the BadiRate CWP-Av method showed the best performance. In the Exp. 2.2, we found that the power of the LRT to detect the simulated rate heterogeneity is highly compromised at low rates (4.5% of the simulations had significant LRT in the *BRlow5* scenario and 10% in the *BRlow10*, whereas in *BRhigh5* and *BRhigh10* scenarios, the percentages were 32% and 84%, respectively). When a different rate in the foreground branch was detected by the LRT, all methods performed relatively well (with some outliers, though) at detecting the direction and magnitude of the rate difference in the foreground species, that is, δ = 10 × β in the foreground species in the *BRhigh10* simulations (fig. 4 and supplementary figs. S6 and S7, Supplementary Material online).

### Global BD Rates in Chemosensory Gene Families

**Fig. 5.—:**
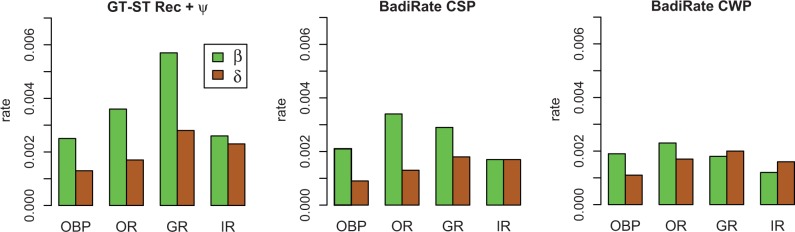
Estimates of global gene birth (β) and death (δ) rates per gene family (OBP, OR, GR, and IR), obtained with three alternative OG-based methods and the exclusion of *Drosophila sechellia*. Estimates obtained with the GT-ST Rec + ψ methods use pseudogene information for all the families except ORs.

In this section, we were interested in comparing the turnover rates of the chemosensory gene families across the *Drosophila* phylogeny and, therefore, we focused on the global rate estimates obtained after the exclusion of *D. sechellia* (fig. 5 and supplementary table S5, Supplementary Material online). In accordance with the results of the simulation experiments, these estimates were more similar among methods than the ones obtained before excluding *D. sechellia* (supplementary table S5, Supplementary Material online). Nevertheless, some discrepancies are still observed among methods, with the BadiRate BD-GR-ML method usually showing the highest estimates and the automated OG-based methods the lowest (supplementary table S5, Supplementary Material online). Overall, the estimates were much closer to the low rates considered in the simulation experiments (0.002) and, therefore, we based our conclusions on the OG-based methods, which have shown the best performance at this rate magnitude (fig. 5). The empirical data allowed us to use pseudogene information (pseudogene sequences were not available for the OR data set). The inclusion of these data in the GT-ST Rec analyses (GT-ST Rec + ψ method) allowed us to take into account duplication events that were posteriorly lost (via pseudogenization), which, as observed, increased both the β and δ estimates (supplementary table S5, Supplementary Material online). This increase, however, was neither substantial nor accompanied by a change in the ratio between β and δ, and probably compensated the slight tendency of the GT-ST Rec method to underestimate rates as observed in the simulation study (supplementary fig. S4, Supplementary Material online).

Gene turnover rates varied among chemosensory gene families. The GT-ST reconciliation methods revealed the GRs as the most dynamic family in terms of gene gains and losses, and the OBPs as the least dynamic. The IR family provides an interesting example of within-family gene turnover rate variation. This family comprises two subfamilies (Croset et al. 2010) with different biological functions. The antennal IRs, which have a role in olfaction (Benton et al. 2009; Croset et al. 2010), were much less dynamic, exhibiting similar gene gain and loss rates that were at least ten times lower than those estimated for the other chemoreceptor families (GT-ST Rec + ψ: β = 0.0001 and δ = 0.0002; supplementary table S5, Supplementary Material online). Conversely, the divergent IRs showed much higher turnover rates, which were comparable to those of the other families analyzed herein (GT-ST Rec + ψ: β = 0.0037 and δ = 0.0032).

Analyses based on OG information indicate differences between β and δ in most of the chemosensory gene families. The ratio between β and δ obtained with the GT-ST reconciliation methods was larger than 1 in the OBP, OR, and GR families, suggesting a putative family expansion in *Drosophila* (fig. 5 and supplementary table S5, Supplementary Material online). The IR family, on the other hand, had very similar β and δ values (constant size) or slightly higher δ than β (reduction trend), depending on the method. We assessed whether the observed differences between β and δ estimates were statistically significant comparing the likelihoods of the BadiRate BD-GR-ML (in which β and δ are allowed to have different values) and BadiRate L-GR-ML (in which β and δ are assumed to be equal) models using the LRT and AIC. We applied this test to the chemosensory gene families and did not find support for differences between β and δ in any of the families (supplementary table S9, Supplementary Material online). Nevertheless, the power of this test to detect differences between β and δ in the *Drosophila* chemosensory gene families has not been evaluated, which is particularly important since, as we mentioned earlier, the simulation experiments demonstrated that the BadiRate BD-GR-ML method might produce highly biased rate estimates.

### Faster Evolutionary Rates in Recently Duplicated Genes

**Fig. 6.—:**
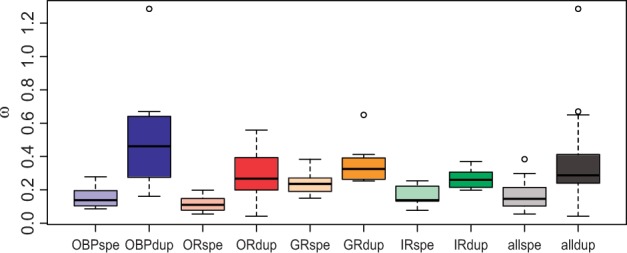
Boxplot summarizing the estimates of ω in speciation (spe) and duplication (dup) branches separately per chemosensory family (OBP, OR, GR, and IR) and the total across families (all).

We investigated the selection regime on recently duplicated chemosensory genes in 38 OGs containing one or more duplicated genes: 9 GRs, 15 ORs, 7 IRs, and 7 OBPs. Of the 38 OGs analyzed, 33 exhibited higher ω estimates in duplication branches (mean = 0.349, SD = 0.213) than in speciation branches (mean = 0.160, SD = 0.075) (fig. 6 and supplementary table S10, Supplementary Material online). This difference in ω was statistically significant (*P* < 0.05) in 25 of these cases and remained significant in 19 cases after the multiple test correction. Remarkably, none of the ω differences was significant at α = 0.05 for the five OGs with the inverse trend (higher ω in speciation branches than in duplication branches) (supplementary table S10, Supplementary Material online).

As expected for functional genes, most of the ω estimates were below 1, indicating that most codons have been evolving under purifying selection. Nevertheless, this analysis estimates a global ω across all sites of a gene, perhaps causing putative positively selected sites in a background of sites mostly evolving under purifying selection to be overlooked. Indeed, it has been shown that some genes with 0.6 < ω < 1 may carry a few positively selected sites (e.g., Swanson et al. 2001; Almeida and DeSalle 2008). We thus tested the hypothesis that at least a few sites are evolving under positive selection in the duplicated branches of OGs with only one duplication event and ω > 0.6: OBP58b and GR47b, with gene duplications in the *D. grimshawi* lineage, and OBP56de and GR64cd, with duplications in the lineage leading to the *Sophophora* subgenus. The statistical tests supported the hypothesis that positive selection is acting on the duplicated branches in all the OGs tested (*P* < 0.01), except for OBP58b (table 3). In the OBP58b OG, the duplicated branches were very short, possibly reducing the statistical power of the selection test.

**Table 3:** Results of Tests for the Presence of Positively Selected Sites on Duplication Branches in Four OGs

OG	ln *L* Selection	ln *L* Null	LRT	ω dup	BEB 95%	ω2f	pω2f
OBP56de	−3,499.4	−3,509.0	19.2*	1.26	5	>1	0.17
GR64cd	−9,792.9	−9,798.3	10.8*	>1	6	>1	0.15
GR47b	−9,823.8	−9,829.4	11.1*	0.65	5	4.81	0.17
OBP58b	−4,120.2	−4,120.3	0.2	0.67	0	n.a.	n.a.

Note.—ω dup, average ω of duplicated branches in the BM; BEB 95%, number of sites with 95% or higher probability of being under selection according to Bayes Empirical Bayes analysis; ω2f, ω of the site class with highest ω; pω2f, frequency of selected sites. *Significant with *P* < 0.01.

## Discussion

Our results uncovered important differences in BD rate estimates obtained with different methods. Full maximum-likelihood methods are straightforward and faster because they are fully automated and do not require knowledge of orthologous relationships (previous identification of OGs) or gene trees and rely solely on the species tree and total gene count per family per species. For these reasons, such methods are more practical at a genomic scale. Moreover, the maximum-likelihood framework allows hypothesis testing, such as the statistical comparison of gene turnover rates between species with different ecological characteristics shown here. Nonetheless, these methods may produce unreliable results due to convergence and local optima problems, as illustrated by the simulation analyses. These problems are particularly relevant in small-sized families and/or when using rich-parameter models (Librado et al. 2012). Our simulation experiments showed that the BadiRate BD-GR-ML method is especially inaccurate when global rates are obtained in the presence of significant rate heterogeneity among lineages, showing thus the importance of testing for rate heterogeneity before calculating global rate estimates with the full likelihood methods in conditions (family characteristics and species relationships) similar to those studied here.

Methods that rely on the reconstruction of the evolutionary history of OGs (GT-ST reconciliation methods), despite being more time consuming and difficult to implement using software (although some programs are available, such as the one described in Dufayard et al. [2005]), benefit from additional information provided by the gene tree. However, their reliability is compromised by limitations in the gene tree reconstruction step, resulting from different evolutionary rates between gene copies and lineages, few variable sites, incomplete lineage sorting, and gene conversion (Hahn 2007; Rasmussen and Kellis 2007). In this study, we attempted to control for some of these potential sources of bias while implementing the GT-ST reconciliation methods. We did detect some cases of gene conversion, but very few of them appeared to have affected the chemosensory gene trees and, consequently, the BD estimates. We certainly did not observe the putative high bias effect on GT-ST Rec estimates predicted by Hahn (2007), such as a 7-fold overestimate of losses compared with the estimates obtained with methods that do not rely on gene trees. In fact, the simulation-based experiments suggest that the GT-ST Rec method is the most accurate and precise in estimating BD rates of the *Drosophila* chemosensory families. The simulation method we used, however, does not take into account problems in gene tree reconstruction (the simulated gene trees are assumed to be accurate). Nevertheless, the similarity between the estimates obtained with the GT-ST Rec method and the automated, OG-based methods for the chemosensory gene families suggests that gene tree reconstructions were not severely biased. Other limitations of the GT-ST Rec method include the lack of an evolutionary model and of a statistical framework that allow hypothesis testing.

In between the GT-ST Rec and the fully automated, maximum likelihood-based method (BadiRate ML) are the automated, parsimony-based methods that use OG information. These methods require delimitation of the OGs prior to the automated analysis, but are not nearly as time-consuming as the GT-ST reconciliation. According to the simulations, the BadiRate CSP and BadiRate CWP behaved similarly to the GT-ST Rec method at rates similar to those observed in the chemosensory gene families. Their slight tendency to produce underestimates in this case was expected given the nature of the parsimony approach. Here, we introduced novel equations to estimate birth and death rates following duplication and loss reconstructions that can be used with both GT-ST reconstruction (GT-ST Rec-Av) and automated methods based on OGs (e.g., BadiRate CWP-Av). These equations were also evaluated in the simulation experiments and proved to be more accurate than the Vieira et al. (2007) equations in the simulations based on high turnover rates (which are far from the observed in chemosensory families), but had less precision in general. It should be noticed that our simulation experiments covered a very small space of empirical possibilities and therefore their results apply only to families very similar to the ones analyzed herein. However, our results reveal the need of further evaluation of the available methods to estimate gene turnover rates in scenarios different from the ones evaluated here.

### Evolution of Size in Chemosensory Gene Families

Herein, we present the first comprehensive comparative study of gene duplication and loss dynamics among the major chemosensory gene families of *Drosophila* by analyzing the same species and using the same analytical methods. The estimated empirical rates were all closer to the simulated low rate (0.002) than to the high rate (0.02), in which case the OG-based methods had in general higher precision and accuracy. The β and δ estimates obtained with these methods were, in most cases, different from those previously reported, even when the same analytical methods were employed. For instance, the gene birth rate we obtained with the GT-ST Rec method for IRs was approximately three times higher than those previously reported (Croset et al. 2010). In addition to methodology, other sources of incongruence among studies include OG delimitation (for methods that use this information) and species sampling. In any case, as previously found, all the estimates shown here (except for the antennal IRs) were larger than the average obtained across all *Drosophila* gene families (0.0012; Hahn et al. 2007), attesting to the relatively rapid turnover rates of chemosensory gene families.

### Lineage-Specific Turnover Rates and Ecology

Ever since it was first observed that *D. sechellia* has fewer OR and GR genes than its sister species, there has been controversy regarding whether the main determinant of this pattern is its diet specialization or its restricted distribution (endemicity) (McBride 2007; McBride et al. 2007). *Drosophila sechellia* is highly specialized in feeding on the *Morinda* fruit, a source that most drosophilids are unable to utilize due to its toxicity. This extreme diet alteration was accompanied by anatomical, molecular, and behavioral changes in *D. sechellia* with respect to its closest relatives (reviewed in Stensmyr 2009). Indeed, diet specialization may render a larger number of chemosensory genes unnecessary and, therefore, may predictably lower the selective constraints on some of these genes. However, the small population sizes often found in endemic species favors the accumulation of neutral and nearly neutral mutations (Ohta 1993, 1973), which may also lead to gene loss through pseudogenization. Studying OBPs, Vieira et al. (2007) found that, as predicted, specialists (represented by *D. sechellia* and *D. erecta*) typically have lower functional constraints (as measured by ω values). In contrast, Gardiner et al. (2008) found no pseudogenization rate differences between diet specialists and generalists in the OR and GR families but rather found higher pseudogenization rates in endemic species (represented by *D. sechellia* and *D. grimshawi*) compared with nonendemic species.

Our analyses corroborated previous studies with regard to *D. sechellia* having exceptional chemosensory gene turnover rates, which severely affects global rate estimates. Although the BM that favors endemicity as the major determinant of gene family size dynamics was the second best-fitting model in some families, our results argue against this conclusion. *Drosophila sechellia* and the only other endemic species analyzed, *D. grimshawi* (with also a very restricted distribution), presented very different patterns of gene turnover: the estimates for *D. sechellia* indicated markedly high δ rates and null (or close to null) β rates, whereas *D. grimshawi* had less dramatic δ estimates, with β being very similar to δ. The relatively high gene birth rate of *D. grimshawi* may explain the higher pseudogeneization rate previously reported for this species (Gardiner et al. 2008).

The BM that favors specialism as a major determinant of gene turnover rates did not show a good fit to the data used herein; however, it is important to note that the specialism level is not equivalent across the studied species. Although *D. sechellia* is a strict specialist, feeding only on one type of food, *D. erecta* is specialist only for part of the year (Rio et al. 1983), and *D. mojavensis* actually utilizes several species of cactus (Oliveira et al. 2012). Another fact that undermines comparisons between *D. sechellia* and *D. mojavensis* (as specialists) and *D. sechellia* and *D. grimshawi* (as endemics) is our taxonomic sampling. *Drosophila sechellia* had a very close relative (*D. simulans*) included in our study, though this did not occur for the other two species. Such a feature of our data prevents a fair evaluation of the factors affecting the BD rates in *D. mojavensis* and *D. grimshawi*; because it is not possible to determine whether their BD rates were similar to or different from those of their closest relatives (unsampled generalist or cosmopolitan species), we cannot determine whether the rate changes correlate with ecological shifts, as in *D. sechellia*. Evidently, we cannot exclude the possibility that endemism and specialism acted synergistically in *D. sechellia* to reduce its repertoire of chemosensory genes because both ecological factors may, in fact, cause higher gene loss rates.

### Gene Turnover Rates and Functional Constraints

Our results suggested that, as a general trend, gene turnover rates were roughly correlated with gene functional constraints. GR, the most dynamic gene family studied, also exhibited the highest protein evolution rates (GT-ST Rec methods), as measured by ω (Sánchez-Gracia et al. 2009). Accordingly, the most dynamic OG groups (with the most gain and loss events) were typically present in the GR subgroups with the highest mean ω (McBride 2007). This was also the case of IRs. It had been shown that divergent IRs exhibit higher rates of protein evolution than antennal IRs (Croset et al. 2010), which have high levels of sequence conservation across insect lineages. Accordingly, we found that divergent IRs are considerably more dynamic in terms of gene turnover than antennal IRs. Interestingly, there is evidence that a few divergent IRs work as gustatory receptors in *Drosophila* (Croset et al. 2010), which suggests that high gene turnover and amino acid substitution rates are hallmarks of gustatory genes.

Remarkably, the differences in the β and δ estimates across methods did not only concern the magnitude but also differentially affected β and δ. Additional simulation experiments would be necessary to determine the accuracy of each method when birth and death rates are dissimilar and the power of these methods to detect differences between these rates. The GT-ST Rec method pointed to an expansion trend in the GRs, OBPs, and ORs when *D. sechellia* is excluded from the analysis. Conversely to our results, McBride and Arguello (2007) found a contraction trend in both ORs and GRs. Nevertheless, they analyzed only the *D. melanogaster* group and, thus, their results could be explained by the extensive gene loss occurring in *D. sechellia* (ORs and GRs) and *D. erecta* (GRs) (supplementary fig. S1 and table S6, Supplementary Material online). According to our estimates, the IR family differed from the other families in that birth and death rates were much more similar to each other. This is an interesting result because the divergent IRs are largely lineage-specific, as are the genes of the other families, which has been interpreted as a sign of family expansion (Croset et al. 2010).

Our approach to OG delimitation was straightforward and rapid in most cases. One exception was the finding of four OBP OGs that were apparently present only in the *D. melanogaster* group, which would imply a high number of convergent, independent gene losses in the remaining *Drosophila* species analyzed. Interestingly, upon further research, we found that the *D. melanogaster* orthologs of these groups are mainly expressed in the male accessory glands instead of in the antennae, as most OBPs. This extreme functional change was likely accompanied by high rates of protein evolution, particularly because the genes expressed in the accessory glands of *Drosophila* species are often under strong positive selection (e.g., Swanson et al. 2001; Almeida and DeSalle 2008). Accordingly, the highest across-species ω value observed among OBPs was in three of these OGs (OBP56i, ω = 0.60; OBP56f, ω = 0.43; and OBP22a, ω = 0.49); the ω estimate of the fourth group (OBP51a; ω = 0.24), though not as high, was also above the OBP average (ω = 0.15; Vieira et al. 2007). The high substitution rates in these four OGs provide a possible explanation for our failure to trace their evolutionary history. These OGs have likely originated from other OBPs through gene duplications and have become so divergent due to their accelerated evolutionary rates that their paralogous relationship cannot be recovered.

### Selective Pressure on Duplicated Genes

Gene duplication is believed to promote functional diversification by allowing relaxed evolution with regard to one or both of the duplicated copies for some period of time after the duplication event (Ohno 1970; Kondrashov et al. 2002). Although some copies may accumulate deleterious mutations and eventually cease to be functional (becoming pseudogenes), others may acquire, by chance, beneficial mutations and evolve under positive selection. As theoretically predicted, there is evidence in the literature that recently duplicated OR and GR genes may have relaxed selective constraints. Guo and Kim (2007) estimated ω between genes within OR OGs and showed that comparisons between paralogous copies often produce larger ω values than those observed in between-orthologs comparisons. In agreement with these results, Gardiner et al. (2008) found that the average ω estimates across genes were significantly higher in OGs with gene duplication than in OGs without duplicates in both the OR and the GR gene families.

Here, we applied for the first time a BM approach to statistically test whether the increased ω values observed in OGs with duplications were, indeed, caused by relaxation of the selective constraints on recently duplicated genes. As predicted, ω was significantly larger between paralogs in most of the OGs tested, including OGs belonging to all the analyzed chemosensory families. Although in most OGs the increases in ω in duplicated branches were likely mainly due to relaxation of the purifying selection, we found evidence that paralog divergence was driven by positive selection in three OGs (one OBP and two GRs). Similarly, but using a slightly different approach, Smadja et al. (2009) inferred the action of positive selection in the differentiation of certain OR and GR paralogs in the pea aphid. These results are important because they suggest that the BD process is not fully stochastic (random genomic drift; Nei 2007) but rather is also clearly influenced by selection, which may act toward the maintenance of at least some gene duplicates (Sánchez-Gracia et al. 2009). Positive selection may, thus, explain some of the gene expansions observed in particular species or lineages.

## Conclusions

Our comprehensive comparative approach to studying chemosensory gene family evolution has shown that the estimation of BD rates is not a simple task and that the methods currently available may produce incongruent results. The simulation experiments showed that estimating global BD rates ignoring the distinctive gene death rate of *D. sechellia* may lead to error, especially when using maximum-likelihood methods. Nevertheless, the maximum-likelihood approach was useful to detect such rate differences and thus to guide further analyses. The GT-ST Rec method to estimate BD rates appeared to be fairly robust in the presence of the small amount of gene conversion and the rate heterogeneity detected in the chemosensory gene families. Automated methods that also employ OG information (such as the BadiRate CSP) performed almost as well as the GT-ST Rec method in the simulation analyses. If our findings apply to other families, these automated and, hence, more practical methods could be good alternatives in comparative studies involving many families (providing that their tendency to underestimate rates is taken into account). Notably, our simulation experiments call attention to the need of more detailed simulation studies to evaluate these methods in scenarios not explored herein. One important aspect that remains to be evaluated is the power of these methods to detect differences between gene birth and death rates and the correct direction of such differences. The GT-ST Rec method suggests that the OBP, OR, and GR families are expanding in *Drosophila*, but this conclusion depends on further analyses. Finally, our findings suggest that the BD process in the studied families is not as stochastic as generally suggested, being rather affected by positive selection on some duplicated genes and certain ecological characteristics of the species.

## Supplementary Material

Supplementary methods, figures S1–S7, and tables S1–S10 are available at *Genome Biology and Evolution* online (http://www.gbe.oxfordjournals.org/).

Supplementary Data
