# Supplementary material for: Family Size Evolution in Drosophila Chemosensory Gene Families: A Comparative Analysis with a Critical Appraisal of Methods
Source: Genome Biol Evol. 2014 Jun 19;6(7):1669–82. doi: 10.1093/gbe/evu130 (PMC4122928; doi:10.1093/gbe/evu130)
Supplement: Supplementary Data [file supp_6_7_1669__index.html]

Family Size Evolution in Drosophila Chemosensory Gene Families: A Comparative Analysis with a Critical Appraisal of Methods — Supplementary Data 

# Family Size Evolution in *Drosophila* Chemosensory Gene Families: A Comparative Analysis with a Critical Appraisal of Methods

## Supplementary Data

file

**Files in this Data Supplement:**

- Supplementary Data - pdf file
- Supplementary Data - pdf file
- Supplementary Data - pdf file
