# Supplementary material for: Family Size Evolution in Drosophila Chemosensory Gene Families: A Comparative Analysis with a Critical Appraisal of Methods
Source: Genome Biol Evol. 2014 Jun 19;6(7):1669–82. doi: 10.1093/gbe/evu130 (PMC4122928; doi:10.1093/gbe/evu130)
Supplement: Supplementary Data [file supp_evu130_Supplementary_Figures.pdf]

Supplementary Figures

Figure S1. *Drosophila* tree showing the number of gene gains and losses per branch and per chemosensory family as estimated using GT-ST reconciliation. The right panel shows the number of genes per species and gene family.

Figure S2. Error in gene birth (β) and death (δ) rate estimates over 500 simulations of experiment 1, as obtained with different computational methods (see text for details). Note that graphs have different scales.

Figure S3. Error in global birth (β) and death (δ) rate estimates obtained with different methods (see text for details) across 10 simulated gene families obtained with the HyPhy library of R. In *GRlow* simulations β=δ=0.002 and in *GRhigh* simulations β=δ=0.02.

Figure S4. Global birth (β) and death (δ) rate estimates obtained with different methods (see text for details) across 10 simulated gene families obtained with the HyPhy library of R. In *GRlow* simulations β=δ=0.002 and in *GRhigh* simulations β=δ=0.02. Red line marks the expected (simulated) values. See main text for details.

Figure S5. Error in gene birth (β) and death (δ) rate estimates over the 500 simulations of experiment 2.1, as obtained with different computational methods (see text for details). Note that graphs have different scales.

Figure S6. Birth (β) and death (δ) rate estimates for the foreground species across the simulated gene families of Exp 2.2 that showed significant rate heterogeneity among

lineages. Red lines mark the expected (simulated) value of the death rate and the blue lines mark the expected value of the birth rate. Outliers of the *BRlow* simulations were excluded from the graph. See main text and Table 1 for details.

Figure S7. Global birth (β) and death (δ) rate estimates for the background species across the simulated gene families of Exp 2.2 that showed significant rate heterogeneity among lineages. Red line marks the expected (simulated) values. See main text and Table 1 for details.

Fig. S1

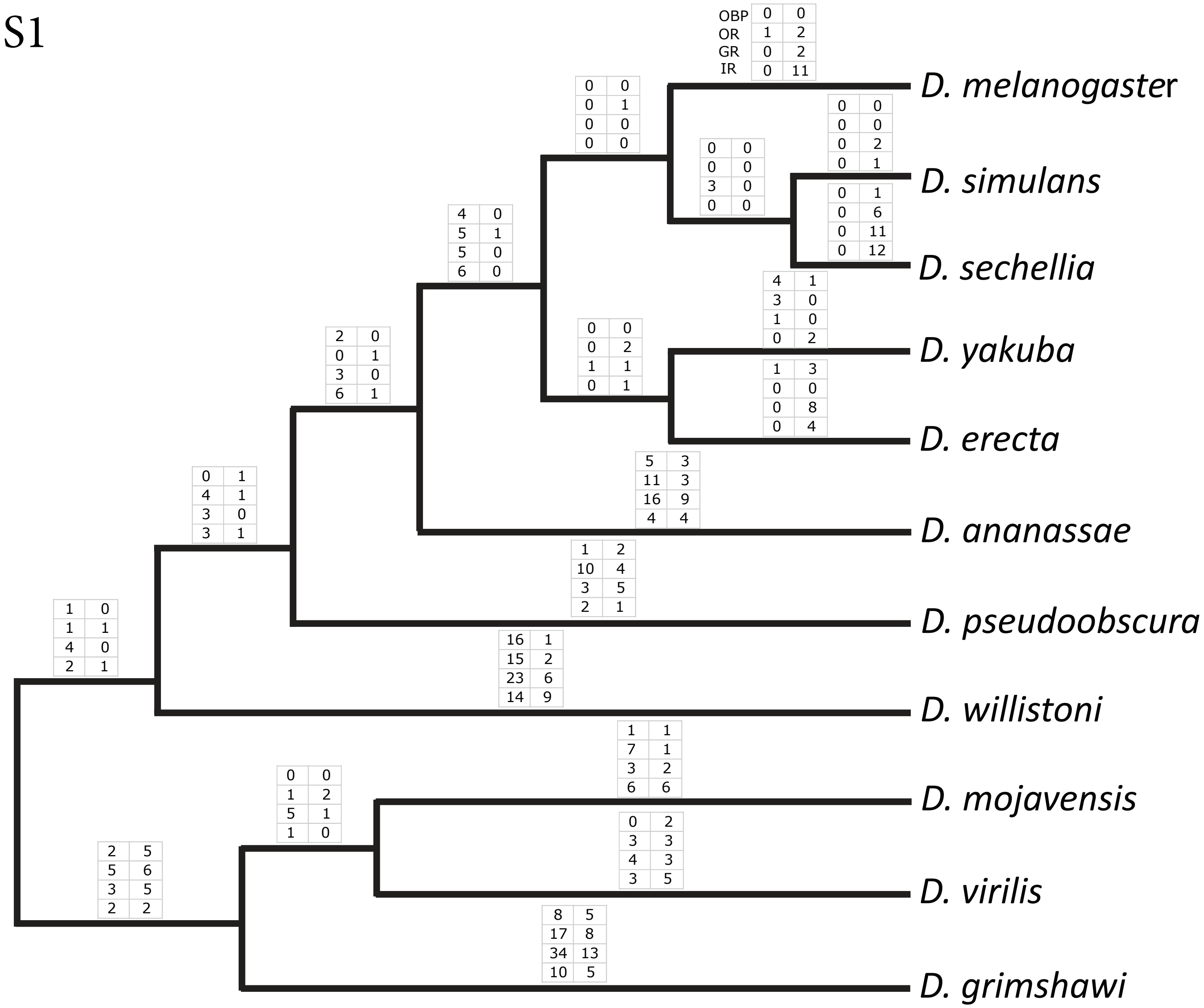

	OBP	OR	GR	IR all	IR div.	Total
D. melanogaster	52	62	60	57	40	231
D. simulans	52	63	62	67	50	242
D. sechellia	51	57	53	56	40	215
D. yakuba	55	65	63	65	48	248
D. erecta	50	62	54	63	46	229
D. ananassae	50	68	64	62	46	244
D. pseudoobscura	45	67	52	58	41	223
D. willistoni	62	71	68	60	43	263
D. mojavensis	43	62	50	56	38	210
D. virilis	41	56	50	54	37	198
D. grimshawi	46	66	66	62	45	244

Fig. S2

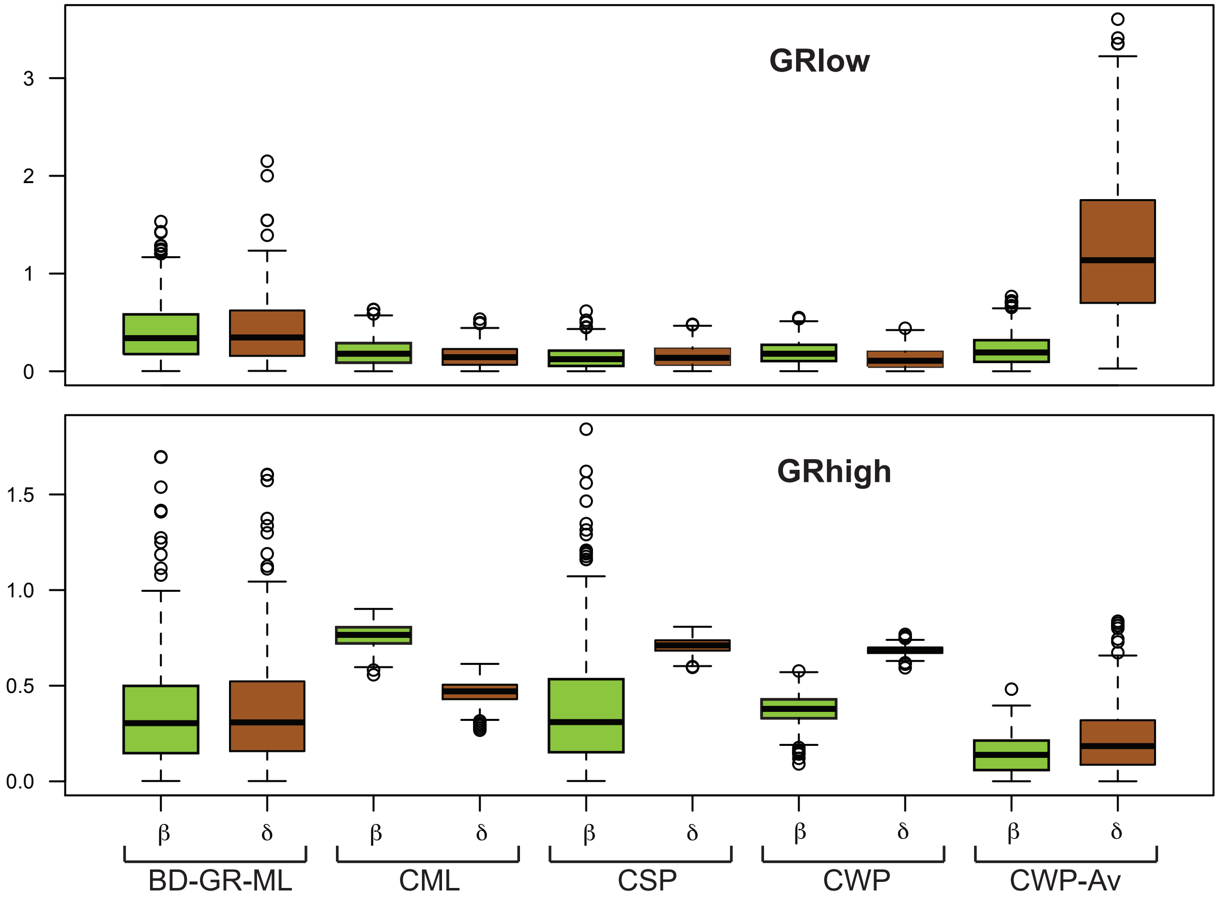

Fig. S3

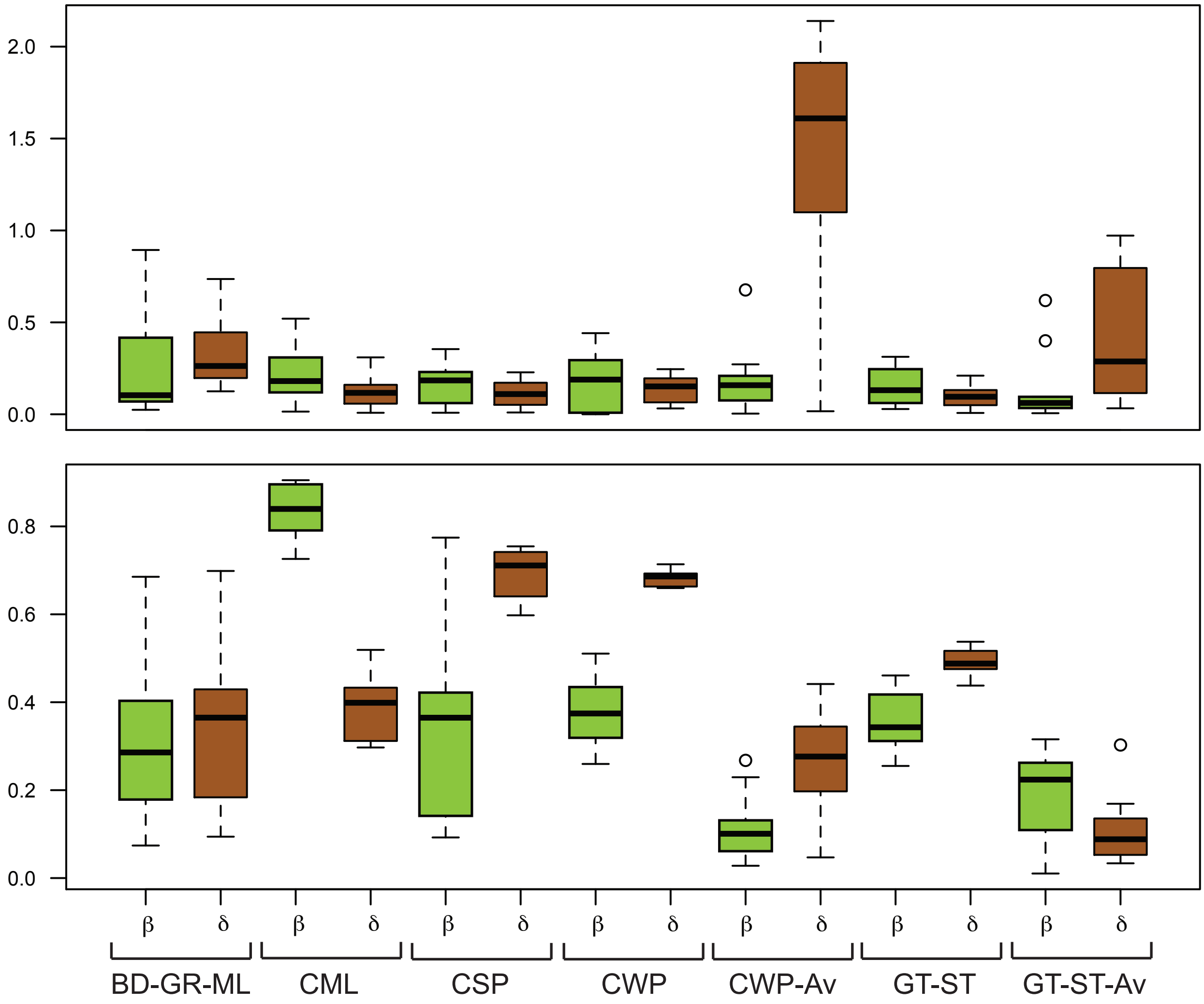

2.0
1.5
1.0
0.5
0.0
0.8
0.6
0.4
0.2
0.0
β
δ
BD-GR-ML
CML
CSP
CWP
CWP-Av
GT-ST
GT-ST-Av

Fig. S4

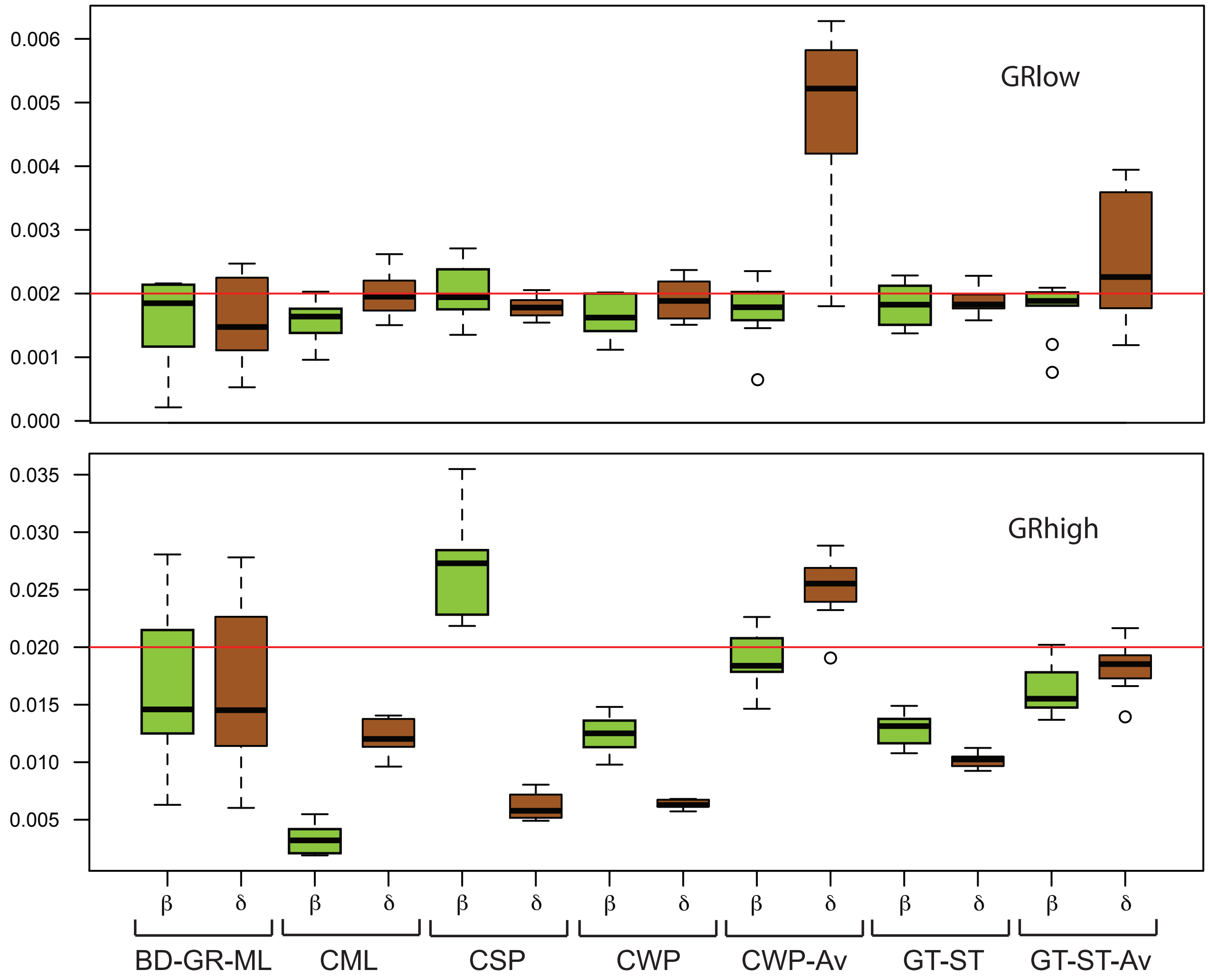

0.006
0.005
0.004
0.003
0.002
0.001
0.000
GRlow
0.035
0.030
0.025
0.020
0.015
0.010
0.005
GRhigh
β δ β δ β δ β δ β δ β δ β δ
BD-GR-ML
CML
CSP
CWP
CWP-Av
GT-ST
GT-ST-Av

Fig. S5

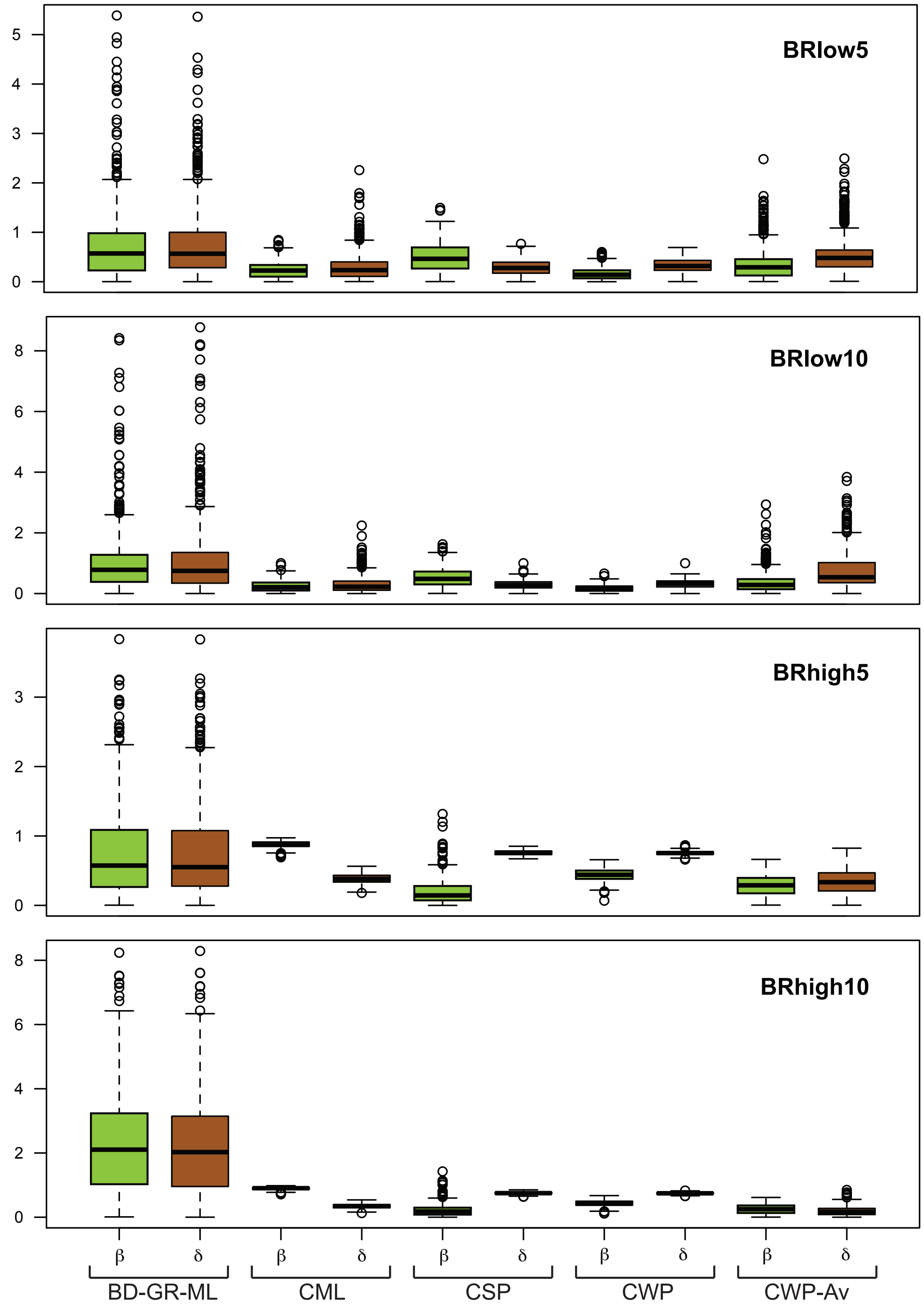
BRlow5
BRlow10
BRhigh5
BRhigh10
0
1
2
3
4
5
6
8
β
δ
BD-GR-ML
CML
CSP
CWP
CWP-Av

Fig. S6

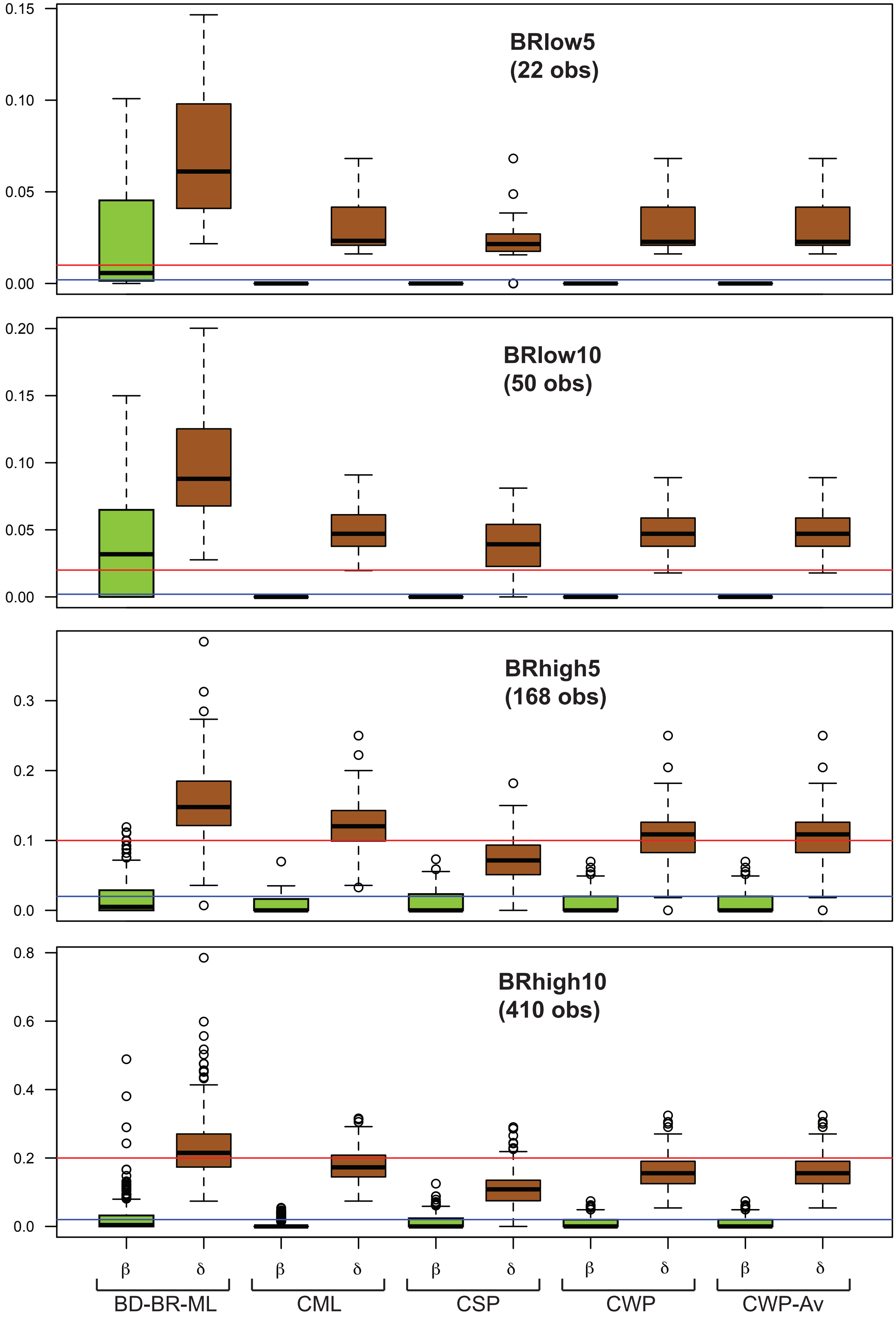

BRlow5
(22 obs)
0.15
0.10
0.05
0.00
BRlow10
(50 obs)
0.20
0.15
0.10
0.05
0.00
BRhigh5
(168 obs)
0.3
0.2
0.1
0.0
BRhigh10
(410 obs)
0.8
0.6
0.4
0.2
0.0
β
δ
BD-BR-ML
CML
CSP
CWP
CWP-Av

Fig. S7

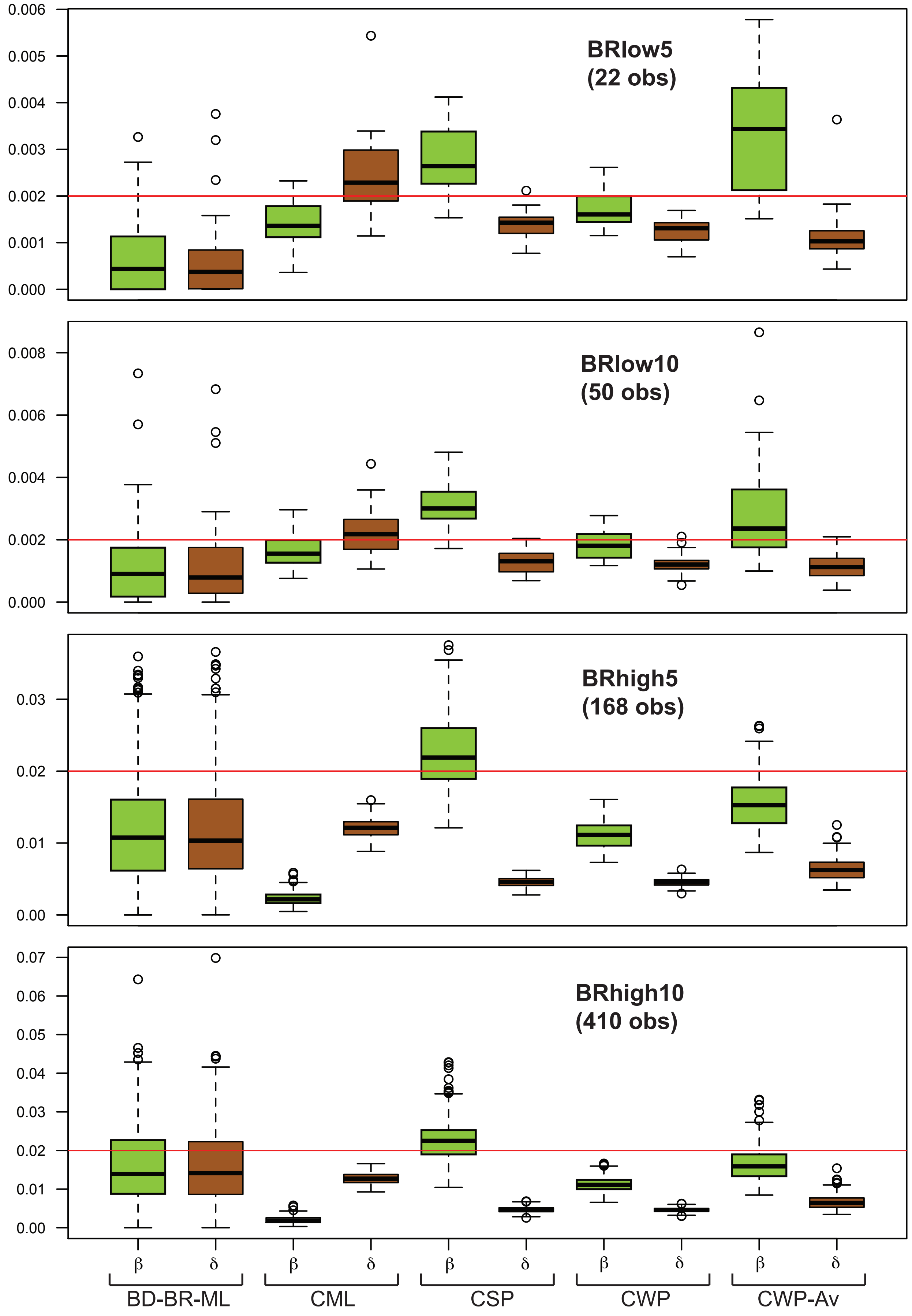

BRlow5
(22 obs)
BRlow10
(50 obs)
BRhigh5
(168 obs)
BRhigh10
(410 obs)
0.006
0.005
0.004
0.003
0.002
0.001
0.000
0.008
0.006
0.004
0.002
0.000
0.03
0.02
0.01
0.00
0.07
0.06
0.05
0.04
0.03
0.02
0.01
0.00
β
δ
BD-BR-ML
CML
CSP
CWP
CWP-Av
