# Supplementary material for: Family Size Evolution in Drosophila Chemosensory Gene Families: A Comparative Analysis with a Critical Appraisal of Methods
Source: Genome Biol Evol. 2014 Jun 19;6(7):1669–82. doi: 10.1093/gbe/evu130 (PMC4122928; doi:10.1093/gbe/evu130)
Supplement: Supplementary Data [file supp_evu130_Supplementary_Methods.pdf]

Supplementary Methods

BadiRate commands

BadiRate CSP:

> perl BadiRate.pl -treefile examples/droso.11sp.tamura.nwk -sizefile OR_OG.txt -out OR_OG_BD_CSP.out -ep CSP –family –rmodel BD

BadiRate CML:

> perl BadiRate.pl -treefile examples/droso.11sp.tamura.nwk -sizefile OR_OG.txt -out OR_OG_BD_CML.out -ep CML –family –rmodel BD

BadiRate CWP:

> perl BadiRate.pl -treefile examples/droso.11sp.tamura.nwk -sizefile OR_OG.txt -out OR_OG_BD_CWP.out -ep CWP –family –rmodel BD

BadiRate BD-GR-ML:

> perl BadiRate.pl -sizefile OR_all.txt -treefile droso.11sp.tamura.nwk -out OR_BD_ML.out -ep ML -rmodel BD -root_dist 0 -start_val 1 -seed $seed

where $seed is a random number obtained with a random number generator.

BadiRate L-GR-ML:

> perl BadiRate.pl -sizefile OR_all.txt -treefile droso.11sp.tamura.nwk -out OR_BD_ML.out -ep ML -rmodel L -root_dist 0 -start_val 1 -seed $seed

BadiRate BD-BR-ML (*Msec*):

> perl BadiRate.pl -sizefile OBP_TIP.txt -treefile droso.11sp.tamura.nwk -start_val 1 -seed $seed -root_dist 0 -rmodel BD -bmodel "4->2_5->1:17->16:4->3:20->18:11->10:14->13:8->6:14->12:22->21:20->19:8->7" -out OBP_GRmodel_replicate$run.txt

Simulation experiments

In Exp. 1, we simulated two sets of 500 gene families (replicates) evolving in 10 species with phylogenetic relationships (including branch lengths) identical to the ones among the 11 *Drosophila* species analyzed here minus *D. sechellia* (see Results) and the same BD rates. One set was simulated with low BD rates (*GRlow*, $\beta=\delta=0.002$ events/gene copy/My) and the other one with high BD rates (*GRhigh*, $\beta=\delta=0.02$ events/gene copy/My). Global BD rates were then estimated for each simulated family and compared to the simulated rates. In Exp. 2 each gene family was simulated in 11 species (phylogenetic relationships identical to the 11 *Drosophila* species analyzed here). Internal branches of the species tree were set to have birth and death rates equal to 0.01 (nuisance parameter), while external (background) branches evolved at low or high rates with the exception of one species (foreground; representing *D. sechellia*), which was set to have a death rate either 5 or 10 times the rate of the background species. In this way, a total of four sets of 500 simulated gene families were generated for Exp. 2 (Table 1), with which we assessed the following two scenarios. In Exp. 2.1, we estimated global

BD rates for the external branches, disregarding the rate heterogeneity among lineages. In Exp. 2.2, we first checked whether the simulated rate heterogeneity could be detected by comparing, for each simulated gene family, the likelihood of two models, one with all external species having the same rates and the other with one species (foreground) having distinctive BD rates. For the replicates in which a significant difference between these two models was detected with the AIC, we estimated background and foreground rates separately.

In all experiment, as done with the empirical data, we analyzed each simulation replicate 100 times with the BadiRate BD-GR-ML and BD-BR-ML methods, each time using different starting values. The presented estimates are based on the run with maximum likelihood.

Gene Conversion

In this section we would like to make clear some of the explanations given in the main text on the evaluation of gene conversion. Gene conversion, when it affects the gene tree, makes two distantly related paralog copies look like recent paralogs. For instance, given the species tree below, with species A, B and C:

Tree A

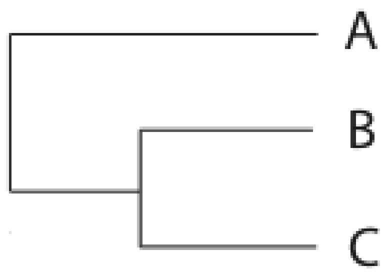

If a duplication happened in the ancestor of species B and C, the gene tree would look like the one below, where numbers designate paralogs within the same species, and the red star shows where duplication happened:

Tree B

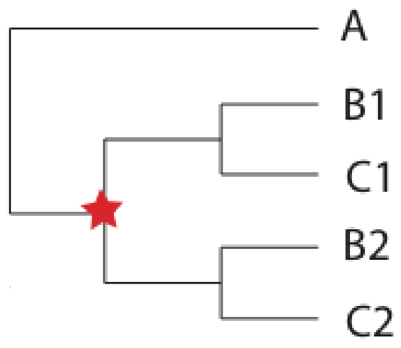

Suppose there is gene conversion between B1 and B2. If the region transferred between paralogs covers a long enough region of the locus, than the affected tree would look like:

Tree C

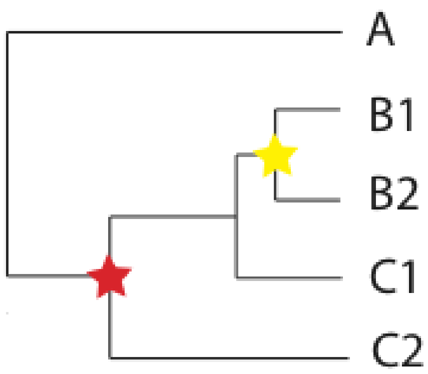

And a duplication would be reconstructed erroneously (yellow star). In this case two duplications would be counted instead of one.

In cases like this, i.e. where gene conversion was detected (using the GENCONV software, see Methods section) and the gene tree looked like Tree C, we corrected the reconstruction to count only one duplication. However, when gene conversion was inferred but the tree looked like the Tree B, we interpreted that tree was neither the gene tree nor the duplication reconstruction were affected.
