# Supplementary material for: Family Size Evolution in Drosophila Chemosensory Gene Families: A Comparative Analysis with a Critical Appraisal of Methods
Source: Genome Biol Evol. 2014 Jun 19;6(7):1669–82. doi: 10.1093/gbe/evu130 (PMC4122928; doi:10.1093/gbe/evu130)
Supplement: Supplementary Data [file supp_evu130_evu130-Supplementary_Tables.pdf]

Supplementary Tables

Table S1. OBP ortholog groups (OG), number of copies per species, total number of gains, losses, and events.

OG	dmel	dsim	dsec	dyak	dere	dana	dpse	dwil	dmoj	dvir	dgri	gains	losses	events
Obp08a	1	1	1	1	1	0	1	1	1	1	0	0	2	2
Obp18a	1	1	0	1	0	1	0	7	1	0	1	6	4	10
Obp19a	1	1	1	1	1	1	1	1	1	1	1	0	0	0
Obp19b	1	1	1	1	1	1	1	1	1	1	1	0	0	0
Obp19c	1	1	1	1	1	1	1	1	1	1	1	0	0	0
Obp19d	1	1	1	1	1	1	1	1	1	1	1	0	0	0
Obp22a	1	1	1	1	2	0	0	0	0	0	0	1	-	1
Obp28a	1	1	1	1	1	1	1	1	1	1	1	0	0	0
Obp44a	1	1	1	1	1	1	1	1	1	1	1	0	0	0
Obp46a	1	1	1	1	1	1	1	1	1	1	1	0	0	0
Obp47a	1	1	1	1	1	1	1	1	1	0	0	0	2	2
Obp47b	1	1	1	1	1	1	1	1	1	1	1	0	0	0
Obp49a	1	1	1	1	1	1	1	1	1	1	1	0	0	0
Obp50a	1	1	1	1	1	1	1	1	1	1	1	0	0	0
Obp50b	1	1	1	1	1	1	1	1	0	0	0	0	1	1
Obp50c	1	1	1	1	1	1	1	3	1	1	1	2	0	2
Obp50d	1	1	1	1	1	1	1	2	0	0	0	1	1	2
Obp50e	1	1	1	1	1	1	1	1	1	1	1	0	0	0
Obp51a	1	1	1	2	0	0	0	0	0	0	0	1	-	1
Obp56a	1	1	1	1	1	1	1	1	1	1	2	1	0	1
Obp56b	1	1	1	1	1	1	1	1	1	1	1	0	0	0
Obp56c	1	1	1	1	1	1	1	1	1	1	1	0	0	0
Obp56de	2	2	2	2	2	2	2	2	3	2	2	3	0	3
Obp56f	1	1	1	1	1	3	0	0	0	0	0	2	-	2
Obp56g	1	1	1	1	1	1	2	5	1	1	1	5	0	5
Obp56h	1	1	1	1	1	1	1	1	1	1	1	0	0	0
Obp56i	1	1	1	1	1	0	0	0	0	0	0	0	-	0
Obp57ab	2	2	2	2	2	1	0	1	0	0	0	1	2	3
Obp57c	1	1	1	1	1	2	1	3	2	2	2	4	0	4
Obp57de	2	2	2	2	2	1	1	1	0	0	0	1	2	3
Obp58b	1	1	1	1	1	1	1	1	1	1	3	2	0	2
Obp58c	1	1	1	1	1	1	1	1	1	1	3	2	0	2
Obp58d	1	1	1	1	1	1	1	1	1	1	1	0	0	0
Obp59a	1	1	1	1	1	0	1	1	1	1	1	0	1	1
Obp69a	1	1	1	1	1	1	1	1	1	1	1	0	0	0
Obp73a	1	1	1	1	1	1	1	1	1	1	1	0	0	0
Obp76a	1	1	1	2	1	1	1	1	1	1	1	1	0	1
Obp83a	1	1	1	1	1	1	1	1	1	1	1	0	0	0
Obp83abL1	0	0	0	0	0	0	0	1	1	1	1	0	1	1
Obp83b	1	1	1	1	1	1	1	1	0	0	0	0	1	1
Obp83cd	1	1	1	1	1	1	1	1	0	1	1	0	1	1
Obp83ef	1	1	1	1	1	1	1	1	1	1	1	0	0	0
Obp83g	1	1	1	1	1	1	1	1	1	1	1	0	0	0
Obp84a	1	1	1	1	1	1	1	1	1	1	1	0	0	0
Obp85a	1	1	1	1	1	1	1	1	1	1	1	0	0	0
Obp93a	1	1	1	2	1	1	1	1	1	1	1	1	0	1
Obp99a	1	1	1	1	1	1	1	1	1	1	1	0	0	0
Obp99b	1	1	1	1	0	3	1	1	1	1	1	2	1	3
Obp99c	1	1	1	1	1	1	1	1	1	1	1	0	0	0
Obp99d	1	1	1	1	1	1	1	1	1	1	1	0	0	0
Total	52	52	51	55	50	50	45	62	43	41	46	36	19	55

Table S2. OR ortholog groups (OG), number of copies per species, total number of gains, losses, and events.

OG	dmel	dsim	dsec	dyak	dere	dana	dpse	dper	dwil	dmoj	dvir	dgri	dups	losses	events
Or01a	1	1	1	1	1	1	1	1	1	1	1	0	0	1	1
Or02a	1	1	1	1	1	1	1	1	1	1	1	0	0	1	1
Or07a	1	1	1	1	1	1	0	0	1	0	0	0	0	2	2
Or09a	1	1	1	1	1	1	1	1	1	1	1	1	0	0	0
Or10a	1	1	1	1	1	1	1	1	1	1	1	1	0	0	0
Or13a	1	1	0	1	1	1	1	1	1	1	0	1	0	2	2
Or19a19b	2	1	1	1	1	1	1	1	1	1	1	1	1	0	1
Or22a	2	2	1	1	1	5	2	2	1	1	1	2	6	2	8
Or22c	1	1	1	1	1	1	1	1	1	1	1	2	1	0	1
Or23a	1	1	1	1	1	1	1	1	1	2	1	1	1	0	1
Or24a	1	1	1	1	1	1	1	1	1	1	1	1	0	0	0
Or30a	1	1	1	1	1	1	1	1	1	1	1	1	0	0	0
Or33a	2	2	2	2	2	5	3	3	1	0	0	0	5	1	6
Or33c	1	1	1	1	1	1	1	1	1	1	1	1	0	0	0
Or35a	1	1	1	1	1	1	1	1	1	1	1	1	0	0	0
Or42a	1	1	1	1	1	1	2	2	1	2	2	1	2	0	2
Or42b	1	1	1	1	1	1	1	1	1	1	1	7	6	0	6
Or43a	1	1	1	1	1	1	1	0	1	1	1	1	0	1	1
Or43b	1	1	1	1	1	1	1	1	1	0	0	0	0	1	1
Or45a	1	1	1	1	1	1	1	1	1	0	0	0	0	1	1
Or45b	1	1	1	1	1	1	1	1	0	1	1	1	0	1	1
Or46a	2	2	2	2	2	2	2	2	2	1	1	3	3	0	3
Or47a	1	1	1	1	1	1	1	1	1	0	0	0	0	1	1
Or47b	1	1	1	1	1	1	1	1	1	2	1	1	1	0	1
Or49a	1	1	1	1	1	1	2	2	1	1	1	2	2	0	2
Or49b	1	1	1	1	1	1	1	1	1	1	1	1	0	0	0
Or56a	1	1	1	1	1	1	1	0	1	1	1	2	1	0	1
Or59a	1	1	1	1	1	1	1	1	2	1	3	3	4	0	4
Or59b	1	1	1	1	1	1	1	1	1	1	2	2	2	0	2
Or59c	1	1	1	1	1	1	1	1	1	0	0	0	0	1	1
Or63a	1	1	1	1	1	1	1	1	1	1	1	1	0	0	0
Or65abc	3	3	2	4	3	3	5	2	2	1	1	0	10	5	15
Or67a	1	2	1	2	1	1	1	0	3	2	2	2	5	1	6
Or67b	1	1	1	1	1	1	1	1	1	1	1	1	0	0	0
Or67c	1	1	1	1	1	1	1	1	1	1	1	1	0	0	0
Or67d	1	1	1	1	1	1	1	1	0	1	1	1	0	1	1
Or67dB	0	0	0	0	0	1	0	0	1	0	0	2	1	3	4
Or69a	2	3	2	3	3	1	2	2	1	1	1	2	3	3	6
Or71a	1	1	1	1	1	1	1	0	1	1	1	1	0	1	1
Or74a	1	1	1	1	1	1	1	1	1	1	1	2	0	1	1
Or82a	1	1	1	1	1	1	1	1	2	1	1	1	1	0	1
Or83a	1	1	1	1	1	1	1	1	1	1	1	1	0	0	0
Or83b	1	1	1	1	1	2	1	1	1	1	1	1	1	0	1
Or83c	1	1	1	1	1	1	1	1	1	1	1	1	0	0	0
Or83c2	0	0	0	0	0	0	0	0	1	1	1	0	0	2	2
Or85a	1	1	1	1	1	0	0	0	1	2	0	0	1	4	5
Or85bc	2	2	2	2	2	2	2	0	1	2	1	1	2	2	4
Or85d	1	1	1	1	1	1	1	1	1	1	1	1	0	0	0
Or85e	1	1	1	1	1	1	1	1	1	1	1	1	0	0	0
Or85f	1	1	1	1	1	1	1	1	6	1	1	1	1	0	1
Or88a	1	1	1	1	1	0	1	1	1	1	1	1	0	1	1
Or92a	1	1	1	2	1	1	1	1	1	1	1	1	1	0	1
Or94a	1	1	1	1	1	1	1	1	1	1	0	1	0	1	1
Or94b	1	1	1	1	1	1	1	0	1	1	1	1	0	1	1
Or98a	1	1	1	2	2	3	3	2	4	3	3	2	8	2	10
Or98b	1	1	0	1	1	1	1	1	1	0	1	0	0	3	3
OrN1	0	0	0	0	0	0	1	0	3	1	1	1	2	2	4
OrN2	0	0	0	0	0	0	0	0	0	5	2	1	5	1	6
Total	62	63	57	65	62	68	67	55	71	62	56	66	76	49	125

Table S3. GR ortholog groups (OG), number of copies per species, total number of gains, losses, and events.

OG	dmel	dsim	dsec	dyak	dere	dana	dpse	dwil	dmoj	dvir	dgri	gains	losses	events
Gr02a	1	1	1	1	1	1	1	1	1	1	1	0	0	0
Gr05a	1	1	1	1	1	1	0	1	1	1	0	0	2	2
Gr08a	1	1	1	1	1	1	1	1	1	1	1	0	0	0
Gr09a	1	1	1	1	1	1	1	1	1	1	1	0	0	0
Gr10a	1	1	1	1	1	1	1	1	1	1	1	0	0	0
Gr10b	1	1	1	1	1	1	1	1	0	0	0	0	2	2
Gr21a	1	1	1	1	1	1	1	1	1	1	1	0	0	0
Gr22a	6	6	3	6	5	4	3	5	1	1	0	8	9	17
Gr23a	1	1	1	1	1	1	1	1	1	1	1	0	0	0
Gr28a	1	1	1	1	1	1	1	1	1	1	1	0	0	0
Gr28b	1	1	1	1	1	1	1	1	1	1	1	0	0	0
Gr32a	1	1	1	1	1	1	1	1	1	1	1	0	0	0
Gr33a	1	1	1	1	1	1	1	1	1	1	1	0	1	1
Gr36abc	3	3	3	2	1	4	1	2	0	0	0	6	3	9
Gr39a	1	1	1	1	1	1	1	1	1	1	1	0	0	0
Gr39b	1	1	0	1	1	1	1	1	1	1	1	0	1	1
Gr43a	1	1	1	1	1	1	1	1	1	1	1	0	0	0
Gr47a	1	1	1	1	0	1	1	1	0	0	1	0	3	3
Gr47b	1	1	1	1	1	1	1	1	1	1	2	1	0	1
Gr57a	1	1	1	1	1	1	1	1	1	1	1	0	0	0
Gr58a	1	1	0	1	1	1	1	1	0	0	0	0	2	2
Gr58b	1	1	1	1	1	1	1	1	1	1	1	0	0	0
Gr58c	1	1	1	1	1	1	1	1	1	1	0	0	1	1
Gr59ab	2	2	3	3	3	4	4	3	1	2	4	10	4	14
Gr59cd	2	3	2	2	2	2	2	8	3	4	7	18	4	22
Gr59e	1	1	1	1	1	1	1	1	1	1	1	0	0	0
Gr59f	1	1	1	1	1	1	1	0	1	1	1	0	1	1
Gr61a	1	1	1	1	1	1	1	1	1	1	2	0	1	1
Gr63a	1	1	1	1	1	1	1	1	1	1	1	0	0	0
Gr64a	1	1	1	1	1	1	1	1	1	1	1	0	0	0
Gr64b	1	1	1	1	1	1	1	1	1	1	1	0	0	0
Gr64cd	2	2	2	2	2	2	2	2	1	1	1	1	1	2
Gr64e	1	1	1	1	1	1	1	1	1	1	1	0	0	0
Gr64f	1	1	1	1	1	1	1	1	1	1	1	0	0	0
Gr66a	1	1	1	1	1	1	1	1	1	1	1	0	1	1
Gr68a	1	1	1	1	1	1	1	1	1	1	1	0	0	0
Gr77a	1	1	1	1	1	1	1	1	1	1	1	0	0	0
Gr85a	1	1	1	2	1	2	1	5	4	5	14	19	12	31
Gr89a	1	1	1	1	1	1	1	1	1	1	2	0	1	1
Gr92a93d	2	3	1	3	0	5	1	0	0	0	0	6	8	14
Gr93a	1	1	1	1	1	1	1	1	1	1	1	0	0	0
Gr93b	1	1	1	1	0	1	1	5	0	0	0	4	3	7
Gr93c	1	1	1	1	1	1	1	1	4	1	2	3	1	4
Gr94a	1	1	1	1	1	1	1	1	1	0	1	0	1	1
Gr97a	1	1	1	1	1	1	1	1	1	1	1	0	0	0
Gr98a	1	1	0	1	1	1	0	1	2	3	2	4	3	7
Gr98bcd	3	3	2	4	3	2	1	1	1	1	1	4	3	7
Total	60	62	53	63	54	64	52	68	50	50	66	84	68	152

Table S4. IR ortholog groups (OG), number of copies per species, total number of gains, losses, and events. *OG*s in italics are the antennal ones.

OG	dmel	dsim	dsec	dyak	dere	dana	dpse	dwil	dmoj	dvir	dgri	gains	losses	events
Ir08a	*1*	*1*	*1*	*1*	*1*	*1*	*1*	*1*	*1*	*1*	*1*	*0*	*0*	*0*
Ir21a	*1*	*1*	*1*	*1*	*1*	*1*	*1*	*1*	*1*	*1*	*1*	*0*	*0*	*0*
Ir25a	*1*	*1*	*1*	*1*	*1*	*1*	*1*	*1*	*1*	*1*	*1*	*0*	*0*	*0*
Ir31a	*1*	*1*	*1*	*1*	*1*	*1*	*1*	*1*	*1*	*1*	*1*	*0*	*0*	*0*
Ir40a	*1*	*1*	*1*	*1*	*1*	*1*	*1*	*1*	*1*	*1*	*1*	*0*	*0*	*0*
Ir41a	*1*	*1*	*1*	*1*	*1*	*0*	*1*	*1*	*1*	*1*	*1*	*0*	*1*	*1*
Ir64a	*1*	*1*	*1*	*1*	*1*	*1*	*1*	*1*	*1*	*1*	*1*	*0*	*0*	*0*
Ir68a	*1*	*1*	*1*	*1*	*1*	*1*	*1*	*1*	*1*	*1*	*1*	*0*	*0*	*0*
Ir75a	*1*	*1*	*0*	*1*	*1*	*1*	*1*	*1*	*1*	*1*	*1*	*0*	*1*	*1*
Ir75b	*1*	*1*	*1*	*1*	*1*	*1*	*1*	*1*	*1*	*1*	*1*	*0*	*0*	*0*
Ir75c	*1*	*1*	*1*	*1*	*1*	*1*	*1*	*1*	*1*	*1*	*1*	*0*	*0*	*0*
Ir76a	*1*	*1*	*1*	*1*	*1*	*1*	*1*	*1*	*1*	*1*	*1*	*0*	*0*	*0*
Ir84a	*1*	*1*	*1*	*1*	*1*	*1*	*1*	*1*	*1*	*1*	*1*	*0*	*0*	*0*
Ir92a	*1*	*1*	*1*	*1*	*1*	*1*	*1*	*1*	*1*	*1*	*1*	*0*	*0*	*0*
Ir93a	*1*	*1*	*1*	*1*	*1*	*1*	*1*	*1*	*1*	*1*	*1*	*0*	*0*	*0*
Ir07a	1	1	1	1	1	1	1	1	1	1	1	0	0	0
Ir07b	1	1	1	1	1	1	1	1	1	1	1	0	0	0
Ir07c	1	1	1	1	1	1	1	1	1	1	1	0	0	0
Ir07d	1	1	1	1	1	1	1	1	1	1	2	1	0	1
Ir07e	1	1	1	1	1	1	1	1	0	0	3	4	4	8
Ir07f	1	1	1	1	1	1	1	1	1	1	1	0	0	0
Ir07g	1	1	1	1	1	1	1	0	0	1	1	0	2	2
Ir10a	0	1	1	1	1	1	1	1	1	1	1	0	1	1
Ir11a	1	1	1	1	1	1	1	1	0	1	0	0	2	2
Ir20a94h	2	2	2	2	2	1	1	1	1	1	1	1	1	2
Ir47a	1	2	0	1	1	1	1	0	1	1	1	1	3	4
Ir48ab	1	2	1	2	1	2	1	1	1	1	1	1	3	4
Ir48c	1	1	1	1	1	0	1	1	0	0	0	0	1	1
Ir48d	0	0	0	0	0	0	1	1	1	1	1	0	1	1
Ir51ab	1	2	2	2	2	2	2	2	1	1	1	2	1	3
Ir52	4	6	4	5	5	4	2	1	3	1	6	10	8	18
Ir54a	1	1	1	1	1	1	1	1	1	1	1	0	0	0
Ir56a	1	1	1	1	1	1	1	1	1	1	1	0	0	0
Ir56b	1	1	1	1	1	1	1	1	1	1	1	0	0	0
Ir56c	1	1	1	1	1	0	1	2	1	1	1	1	1	2
Ir56d	1	1	1	1	1	1	1	2	1	1	1	1	0	1
Ir56e	0	0	0	0	0	0	0	1	1	1	1	0	1	1
Ir60a	1	1	1	1	1	1	1	1	1	1	1	0	0	0
Ir60b	2	3	2	3	4	4	3	2	1	1	1	4	5	9
Ir60e	0	1	1	1	1	1	1	1	1	0	1	0	2	2
Ir62a	1	1	1	1	1	1	1	1	1	1	1	0	0	0
Ir67a	0	1	1	1	1	1	1	1	1	1	1	0	1	1
Ir67b	1	1	1	1	1	1	1	0	1	1	1	0	1	1
Ir67c	1	1	0	1	1	1	1	1	0	0	0	0	1	1
Ir68b	1	1	1	1	1	1	1	2	2	1	1	2	0	2
Ir75d	1	1	1	1	1	1	1	1	2	1	1	1	0	1
Ir76b	1	1	1	1	1	1	1	1	1	1	2	1	0	1
Ir85a	1	1	1	1	1	1	1	1	1	1	1	0	0	0
Ir87a	1	1	1	1	1	1	1	1	1	1	1	0	0	0
Ir94abc	3	4	1	4	2	5	2	3	3	4	1	13	7	20
Ir94d	1	1	1	1	1	1	1	1	0	1	0	0	2	2
Ir94e	1	1	1	1	1	1	1	1	2	1	2	2	0	2
Ir94f	1	1	1	1	1	1	0	2	1	1	2	2	1	3
Ir94g	1	1	1	1	1	1	1	1	1	1	1	0	0	0
Ir100a	1	1	1	1	1	1	1	1	1	1	1	0	0	0
Total	55	65	54	63	61	60	56	58	54	52	60	47	51	98

Table S5. Gene birth and death rate estimates (birth – death) for four chemosensory gene families and two IR subfamilies using different methods

method	OG?[a]	OBP	OR	GR	IR	divergent IR	antennal IR
**Using Vieira et al. (2007) equations**							
GT-ST-Rec+ψ	Y	0.0025 - 0.0014[b]	n.a.	0.0057 - 0.0032	0.0026 - 0.0027	0.0037 - 0.0037	0.0001 - 0.0003
GT-ST-Rec	Y	0.0022 - 0.0011	0.0036 - 0.0019	0.0050 - 0.0026	0.0021 - 0.0023	0.0030 - 0.0031	0.0001 - 0.0003
BadiRate CWP	Y	0.0019 – 0.0011	0.0023 – 0.0018	0.0019 – 0.0023	0.0014 -0.0016	0.0020 – 0.0022	0.0001 - 0.0003
BadiRate CSP	Y	0.0021 - 0.0009	0.0037 - 0.0013	0.0029 - 0.0019	0.0017 - 0.0017	0.0026 - 0.0022	0.0001 - 0.0003
BadiRate CML	Y	0.0021 - 0.0009	0.0021 - 0.0020	0.0036 - 0.0016	0.0019 - 0.0015	0.0029 - 0.0020	0.0001 - 0.0003
**“Branch average” approach**							
GT-ST-Rec-Av+ψ	Y	0.0027 – 0.0021	n.a.	0.0083 – 0.0126	0.0020 – 0.0128	0.0028 – 0.0162	0.0001 – 0.0003
GT-ST-Rec-Av	Y	0.0025 – 0.0020	0.0034 – 0.0076	0.0081 - 0.0123	0.0018 – 0.0126	0.0025 – 0.0159	0.0001 – 0.0003
BadiRate CWP-Av	Y	0.0012 – 0.0022	0.0037 – 0.0066	0.0053 – 0.0110	0.0062 – 0.074	0.0087 – 0.0093	0.0001 – 0.0028
BadiRate BD-GR-ML	N	0.0065 - 0.0052	0.0136 - 0.0132	0.0393 - 0.0372	0.0434 - 0.0442	0.0500 - 0.0511	0.0002 - 0.0003
BadiRate L-GR-ML	N	0.0061	0.0134	0.0398	0.0438	0.0505	0.0003
**Without *D. sechellia***							
GT-ST-Rec+ ψ	Y	0.0025 – 0.0013	0.0036 – 0.0017[c]	0.0057 – 0.0028	0.0026 - 0.0023	0.0037 – 0.0032	0.0001 - 0.0002
GT-ST-Rec-Av+ ψ	Y	0.0030 – 0.0013	0.0038 – 0.0032[c]	0.0091 – 0.0031	0.0022 - 0.0038	0.0031 – 0.0051	0.0001 – 0.0001
Badirate CWP	Y	0.0019 – 0.0011	0.0023 – 0.0017	0.0018 – 0.0020	0.0012 – 0.0016	0.0012 – 0.0016	0.0001 – 0.0001
BadiRate CWP-Av	Y	0.0013 – 0.0014	0.0022 – 0.0039	0.0033 – 0.0051	0.0014 – 0.0043	0.0019 – 0.0061	0.0001 – 0.0001
BadiRate CSP	Y	0.0021 – 0.0009	0.0034 – 0.0013	0.0029 – 0.0018	0.0017 – 0.0017	0.0026 – 0.0022	0.0001 – 0.0001
BadiRate CML	Y	0.0016 – 0.0018	0.0019 – 0.0020	0.0027 – 0.0015	0.0010 – 0.0018	0.0016 – 0.0022	0.0001 – 0.0001
BadiRate BD-GR-ML	N	0.0063 – 0.0050	0.0031 - 0.0024	0.0133 - 0.0110	0.0090 - 0.0096	0.0128 -0.0136	0.0002 – 0.0002

[a] Method relies on ortologous group information? Y= yes, N= no. [b] The values before and after the dash represent the gene birth and death rates, respectively. [c] Pseudogene information was not available and therefore not taken into account.

Table S6. Gene birth (β) and death (δ) rate estimates per branch obtained with the GT-ST Rec-Av method.

branch	gains	losses	genes	gain rate	loss rate	time[a]	β	δ
OBP								
sim	0	0	52	0	0	1	0	0
sec	0	1	51	0	0.0196	1	0	**0.0196**
mel	0	0	52	0	0	5	0	0
(sim, sec)	0	0	52	0	0	4	0	0
(mel,sim,sec)	0	0	52	0	0	8	0	0
yak	4	1	55	0.0727	0.0182	10	0.0073	0.0018
ere	1	3	50	0.0200	0.0600	10	0.0020	0.0060
(yak,ere)	0	0	52	0	0	3	0	0
(8,5)	4	0	52	0.0769	0	31	0.0025	0
ana	5	3	50	0.1000	0.0600	44	0.0023	0.0014
(10,9)	2	0	48	0.0417	0	11	0.0038	0
pse	1	2	45	0.0222	0.0444	55	0.0004	0.0008
(14,11)	0	1	46	0	0.0217	7	0	0.0031
wil	16	1	62	0.2581	0.0161	62	0.0042	0.0003
(wil,15)	1	0	47	0.0213	0	1	0.0213	0
moj	1	1	43	0.0233	0.0233	31	0.0008	0.0008
vir	0	2	41	0	0.0488	31	0	0.0016
(moj,vir)	0	0	42	0	0	12	0	0
gri	8	5	42	0.1905	0.1190	43	0.0044	0.0028
(gri,20)	2	5	42	0.0476	0.1190	20	0.0024	0.0060
OR								
sim	3	1	61	0.0492	0.0164	1	0.0492	0.01639
sec	1	6	55	0.0182	0.1091	1	0.0182	**0.10909**
mel	1	1	62	0.0161	0.0161	5	0.0032	0.00323
(sim, sec)	0	3	59	0.0000	0.0508	4	0	0.01271
(mel,sim,sec)	0	1	62	0.0000	0.0161	8	0	0.00202
yak	3	0	65	0.0462	0.0000	10	0.0046	0
ere	0	0	62	0.0000	0.0000	10	0	0
(yak,ere)	0	1	62	0.0000	0.0161	3	0	0.00538
(8,5)	3	1	63	0.0476	0.0159	31	0.0015	0.00051
ana	10	3	68	0.1471	0.0441	44	0.0033	0.00100
(10,9)	0	1	61	0.0000	0.0164	11	0	0.00149
pse	8	2	64	0.1250	0.0313	55	0.0023	0.00057
(14,11)	4	1	62	0.0645	0.0161	7	0.0092	0.00230
wil	15	1	73	0.2055	0.0137	62	0.0033	0.00022
(wil,15)	2	0	59	0.0339	0.0000	1	0.0339	0
moj	9	1	61	0.1475	0.0164	31	0.0048	0.00053
vir	5	5	53	0.0943	0.0943	31	0.0030	0.00304
(moj,vir)	0	0	53	0.0000	0.0000	12	0	0
gri	23	6	70	0.3286	0.0857	43	0.0076	0.00199
(gri,20)	2	6	53	0.0377	0.1132	20	0.0019	0.00566
GR								
sim	0	2	62	0.0000	0.0323	1	0	0.0323
sec	0	11	53	0.0000	0.2075	1	0	**0.2075**
mel	0	2	60	0.0000	0.0333	5	0	0.0067
(sim, sec)	2	0	64	0.0313	0.0000	4	0.0078	0
(mel,sim,sec)	0	0	62	0.0000	0.0000	8	0	0
yak	1	0	63	0.0159	0.0000	10	0.0016	0
ere	0	8	54	0.0000	0.1481	10	0	0.0148
(yak,ere)	1	1	62	0.0161	0.0161	3	0.0054	0.0054
(8,5)	5	0	62	0.0806	0.0000	31	0.0026	0

ana	16	9	64	0.2500	0.1406	44	0.0057	0.0032
(10,9)	4	0	57	0.0702	0.0000	11	0.0064	0
pse	3	5	52	0.0577	0.0962	55	0.0010	0.0017
(14,11)	3	0	54	0.0556	0.0000	7	0.0079	0
wil	23	6	68	0.3382	0.0882	62	0.0055	0.0014
(wil,15)	4	0	51	0.0784	0.0000	1	0.0784	0
moj	3	2	50	0.0600	0.0400	31	0.0019	0.0013
vir	4	3	50	0.0800	0.0600	31	0.0026	0.0019
(moj,vir)	5	1	49	0.1020	0.0204	12	0.0085	0.0017
gri	34	13	66	0.5152	0.1970	43	0.0120	0.0046
(gri,20)	3	5	45	0.0667	0.1111	20	0.0033	0.0056
IR								
sim	0	1	67	0	0.0149	1	0	0.0149
sec	0	12	56	0	0.2143	1	0	**0.2143**
mel	0	10	57	0	0.1754	5	0	**0.0351**
(sim, sec)	0	0	68	0	0	4	0	0
(mel,sim,sec)	0	0	68	0	0	8	0	0
yak	0	2	65	0	0.0308	10	0	0.0031
ere	0	4	63	0	0.0635	10	0	0.0063
(yak,ere)	0	1	67	0	0.0149	3	0	0.0050
(8,5)	6	0	68	0.0882	0	31	0.0028	0
ana	4	4	62	0.0645	0.0645	44	0.0015	0.0015
(10,9)	6	1	62	0.0968	0.0161	11	0.0088	0.0015
pse	2	1	58	0.0345	0.0172	55	0.0006	0.0003
(14,11)	3	1	57	0.0526	0.0175	7	0.0075	0.0025
wil	13	9	60	0.2167	0.1500	62	0.0035	0.0024
(wil,15)	0	0	55	0	0	1	0	0
moj	6	6	56	0.1071	0.1071	31	0.0035	0.0035
vir	3	5	54	0.0556	0.0926	31	0.0018	0.0030
(moj,vir)	1	0	56	0.0179	0.0000	12	0.0015	0
gri	10	5	60	0.1667	0.0833	43	0.0039	0.0019
(gri,20)	2	2	55	0.0364	0.0364	20	0.0018	0.0018

[a] In million years

Table S7. Results of the branch model analyses of gene birth (β) and death (δ) rates using BadiRate BD-BR-ML. L = likelihood, AIC = Akaike information criterion, int = internal branches, ext = external branches. See main text for description of the models.

All families									
model	np	L	AIC	int β	int δ	ext β	ext δ	focal β	focal δ
MGr	4	-158.12	324.25	0.0005	0.0005	0.0322	0.0317	n.a.	n.a.
Msec	6	-140.10	**292.19**	0.0047	0.0047	0.0092	0.0085	0.0338	0.1453
Mgri	6	-156.33	324.67	0.0020	0.0022	0.0043	0.0026	0.0341	0.0336
Mspe	6	-152.58	317.16	0.0025	0.0021	0.0133	0.0126	0.1139	0.1176
Mend	6	-148.51	309.02	0.0048	0.0049	0.2069	0.2069	0.0093	0.0088
Mspeend	6	-155.82	323.64	0.0015	0.0011	0.0164	0.0159	0.0784	0.0794
Mspe-end-sec	10	-153.38[a]	326.77	0.0117	0.0128	0.0084	0.0072	n.a.	n.a.
MFr	26	[a]							

OBP family									
model	np	L	AIC	int β	int δ	ext β	ext δ	focal β	focal δ
MGr	4	-31.53	71.06	0.0038	0.0032	0.0073	0.0059	n.a.	n.a.
Msec	6	-31.14	74.28	0.0046	0.0039	0.0067	0.0051	0.0000	0.0154
Mgri	6	-30.54	73.09	0.0017	0.0000	0.0000	0.0000	0.0097	0.0084
Mspe	6	-29.52	71.05	0.0019	0.0000	0.0100	0.0081	0.0000	0.0020
Mend	6	-30.48	72.96	0.0016	0.0000	0.0000	0.0001	0.0108	0.0095
Mspeend	6	-29.46	**70.91**	0.0000	0.0000	0.0144	0.0145	0.0000	0.0036
Mspe-end-sec	10	[a]							
MFr	26	[a]							

GR family									
model	np	L	AIC	int β	int δ	ext β	ext δ	focal β	focal δ
MGr	4	-40.84	89.68	0.0014	0.0000	0.0454	0.0434	n.a.	n.a.
Msec	6	-35.19	**82.39**	0.0025	0.0000	0.0150	0.0133	0.0000	0.1442
Mgri	6	-39.27	90.53	0.0025	0.0021	0.0058	0.0000	0.0530	0.0503
Mspe	6	-37.19	86.37	0.0023	0.0000	0.0108	0.0085	0.1796	0.1883
Mend	6	-37.83	87.67	0.0025	0.0000	0.2928	0.2910	0.0145	0.0134
Mspeend	6	-38.46	88.91	0.0024	0.0000	0.0104	0.0085	0.1619	0.1633
Mspe-end-sec	10	[a]							
MFr	26	[a]							

IR family									
model	np	L	AIC	int β	int δ	ext β	ext δ	focal β	focal δ
MGr	4	-41.40	90.80	0.0000	0.0000	0.0502	0.0511	n.a.	n.a.
Msec	6	-33.61	**79.22**	0.0370	0.0367	0.0007	0.0013	0.0000	0.1790
Mgri	6	-39.45	90.89	0.0004	0.0001	0.0000	0.0000	0.0493	0.0497
Mspe	6	-39.61	91.21	0.0005	0.0000	0.0180	0.0199	0.2136	0.2208
Mend	6	-37.43	86.86	0.0408	0.0405	0.4512	0.4548	0.0008	0.0015
Mspeend	*6*	-40.73	93.45	0.0005	0.0000	0.0224	0.0242	0.1634	0.1673
Mspe-end-sec	10	[a]							
MFr	26	[a]							

OR family									
model	np	L	AIC	int β	int δ	ext β	ext δ	focal β	focal δ
MGr	4	-36.30	80.60	0.0161	0.0143	0.0000	0.0002	n.a.	n.a.
Msec	6	-28.84	**69.67**	0.1030	0.1882	0.0029	0.0021	0.0004	0.0014
Mgri	6	-36.42	84.84	0.0145	0.0143	0.0007	0.0000	0.0036	0.0059
Mspe	6	-33.45	78.91	0.2656	0.2663	0.0018	0.0002	0.0038	0.0052
Mend	6	-31.56	75.13	0.0031	0.0022	0.1347	0.1351	0.0007	0.0016
Mspeend	6	-34.42	80.85	0.1762	0.1739	0.0009	0.0000	0.0044	0.0068
Mspe-end-sec	10	[a]							
MFr	26	[a]							

[a] Not converged.

Table S8. Results of the gene conversion analysis.

OG[a]	paralog 1	paralog 2	BC p[b]	length	total length	affect GT[c]?	in tandem
IR60b	DwilIR60m	DwilIR60n	<0.001	187	1840	maybe	yes
	Dper/DpseIR60j	Dper/DpseIR60k	<0.001	327	1840	no	yes
IR7eg	Dgri7e1	Dgri7e3	0.0039	262	2025	maybe	yes
	Dgri7e1	Dgri7e2	0.034	536	2025	maybe	no, but same chr
IR52b	Dere/DsimIR52b	Dere/DsimIR52e	<0.001	146	2028	maybe	yes
	Dper/DpseIR52j	Dper/DpseIR52k	<0.001	355	2028	likely	yes
	DgriIR52q	DgriIR52s	<0.001	381	2028	maybe	yes
	DgriIR52q	DgriIR52r	<0.001	150	2028	maybe	yes
	DmojIR52n	DmojIR52p	0.0018	98	2028	maybe	yes
	DmojIR52o	DmojIR52p	<0.001	230	2028	maybe	yes
IR94az	DperIR94o	DperIR94p	<0.001	241	1995	no	yes
	DvirIR94w	DvirIR94x	0.015	33	1995	no	no, but same chr
IR51ab	Dper/DpseIR51c	Dper/DpseIR51d	<0.001	79	2010	likely	yes
GR59ab	DgriGR59bL2	DgriGR59bL3	<0.001	180	1188	no	yes
	DgriGR59bL1	DgriGR59bL2	<0.001	129	1188	no	yes
	DgriGR59bL1	DgriGR59bL3	<0.001	105	1188	no	yes
	DgriGR59bL3	DgriGR59bL4	<0.001	110	1188	maybe	yes
	DgriGR59bL2	DgriGR59bL4	<0.001	110	1188	maybe	yes
	DperGR59aL4	DperGR59a	<0.001	93	1188	maybe	yes
	DperGR59aL4	DperGR59aL3	<0.001	86	1188	maybe	yes
	DpseGR59aL3	DpseGR59aL4	<0.001	86	1188	maybe	yes
OR22a	DanaOR22a3	DanaOR22a4B	<0.001	465	1206	no	yes
	DanaOR22a3	DanaOR22a4A	<0.001	465	1206	no	yes
	DanaOR22a4A	DanaOR22a4B	<0.001	1080	1206	no	yes
	DsimOR22a1	DsimOR22a2	0.014	110	1206	no	yes
OR65bc	DpseOR65b1	DpseOR65b3	<0.001	212	1290	no	yes
	DpseOR65b2	DpseOR65b3	<0.001	179	1290	no	yes
	DpseOR65b3	DpseOR65b4	0.0057	98	1290	no	yes
	DyakOR65c1	DyakOR65c2	0.0045	197	1290	no	yes
	DereOR65b	DereOR65c	0.039	73	1290	no	yes

[a] The other OGs analyzed and for which no convergence events were detected were: Gr92a93b, Gr36abc, Gr59ab, Gr59cd, Gr22a, Or22a, Or98a, Or65abc, Ir7eg, Ir51ab, Ir52b, Ir60b, Ir94a, and Obp18a. [b] Bonferroni correct *p* value. [c] Gene tree

Table S9. Results of the likelihood-based test for differences between gene birth and death rates.

gene family	lnL L ($\beta = \delta$)	lnL BD ($\beta \neq \delta$)	LRT	AIC L	AIC BD
OBP	-30.397	-30.256	0.28	64.75	64.51
OR	-31.377	-31.335	0.08	64.75	66.67
GR	-35.168	-34.729	0.88	72.34	73.46
IR	-33.698	-33.661	0.07	69.40	71.32

lnL L = log likelihood of model BadiRate L-GR-ML (1 parameter); lnL = loglikelihood of model of model BadiRate BD-GR-ML (2 parameters); LRT = likelihood ratio test results with df = 1 (The critical value for significance at a = 0.05 is 3.84); AIC = Akaike information criterion.

Table S10. Results of tests for differences in evolutionary constraints (ω) between duplication and speciation branches.

OG	lnL M0	lnL BM2	LRT	*p* LRT	adjusted α	ω_{sel}	ω_{dup}	d_S
OR42b	-7803.9	-7689.1	229.5	1.43E-50	0.0013	0.060	0.559	<1
OR59b	-8145.8	-8055.4	180.7	5.84E-40	0.0014	0.055	0.371	<1.54
GR93c	-13059.1	-13032.3	53.4	2.48E-12	0.0014	0.174	0.412	<1.42
GR47b	-9971.3	-9948.2	46.2	9.31E-11	0.0014	0.150	0.650	<1.78
OR49a	-9664.6	-9643.5	42.3	6.57E-10	0.0015	0.168	0.496	<1.2
OR22a	-13577.4	-13558.5	37.7	6.38E-09	0.0015	0.146	0.257	<1.34
OBP99b	-2647.9	-2629.4	37.0	9.46E-09	0.0016	0.086	0.462	<1.48
OR42a	-9592.4	-9574.4	36.1	1.42E-08	0.0016	0.088	0.327	<1.08
GR59ab	-26321.7	-26305.8	31.8	1.25E-07	0.0017	0.231	0.371	<1.33
OR33a	-9805.5	-9792.7	25.5	2.96E-06	0.0017	0.133	0.287	<1.12
OBP56g	-3235.3	-3223.6	23.2	9.13E-06	0.0018	0.175	0.612	<2
IR94e	-13750.3	-13739.4	21.7	1.93E-05	0.0019	0.134	0.260	<1.12
OR74a	-8020.2	-8009.6	21.1	2.59E-05	0.0019	0.096	0.240	<1.2
OR59a	-4793.1	-4784.4	17.4	0.0002	0.0020	0.069	0.164	<1.23
IR94f	-13405.2	-13397.1	16.2	0.0003	0.0021	0.214	0.370	<1
GR64cd	-11569.7	-11562.4	14.6	0.0007	0.0022	0.084	>1	<1.43
OBP58b	-3930.8	-3924.0	13.7	0.0011	0.0023	0.087	0.670	<1.7
IR52	-35368.3	-35361.9	12.8	0.0017	0.0024	0.230	0.289	<1.01
IR75d	-9984.0	-9977.9	12.2	0.0023[a]	0.0025	0.077	0.228	<1.02
IR60	-31071.5	-31065.7	11.6	0.0030	0.0026	0.254	0.324	<1.03
IR68b	-16026.7	-16021.0	11.5	0.0032	0.0028	0.131	0.202	<1.28
OR85c	-8170.1	-8164.8	10.5	0.0053	0.0029	0.125	0.416	<1
OBP56a	-2482.2	-2478.5	7.3	0.0261	0.0031	0.122	0.285	<1
OBP56de	-3520.1	-3516.6	7.0	0.0304	0.0033	0.138	1.286	<1.25
GR98a	-13305.1	-13301.7	6.9	0.0317[b]	0.0036	0.208	0.319	<1.21
OR56a	-1199.2	-1196.3	5.8	0.0548	0.0038	0.103	0.267	<1.35
OR69a	-445.5	-444.0	2.9	0.2306	0.0042	0.061	0.462	<1.22
OR23a	-9142.2	-9140.8	2.9	0.2396	0.0045	0.150	0.210	<1.7
GR92a93d	-14213.0	-14212.3	1.4	0.5016	0.0050	0.384	0.331	<1.84
OBP57de	-3167.2	-3166.5	1.3	0.5205	0.0056	0.278	0.161	<1.32
IR48ab	-15951.8	-15951.2	1.2	0.5498	0.0063	0.138	0.198	<1.58
OR82a	-4261.2	-4260.7	1.1	0.5867	0.0071	0.110	0.138	<1.35
OBP56c	-4986.0	-4985.5	0.9	0.6372	0.0083	0.216	0.266	<1.3
OR85f	-109.5	-109.2	0.8	0.6706	0.0100	0.166	0.042	<1.31
GR36abc	-12667.7	-12667.4	0.6	0.7438	0.0125	0.298	0.272	<1.06
GR98bcd	-16522.9	-16522.8	0.3	0.8663	0.0167	0.240	0.256	<1.23
OR67a	-14670.8	-14670.7	0.2	0.8909	0.0250	0.198	0.188	<1.28
GR59cd	-31916.0	-31915.9	0.2	0.9048	0.0500	0.245	0.253	<1.95

[a] Comparisons above the first line are significant after sequential Bonferroni correction.[b] Comparisons above the second line are significant with α = 0.0
